# Biosocial Factors Shaping Perceptions of Disease Risk Among a Community‐Based Sample of Sexual and Gender Minority People Living in Toronto During the COVID‐19 Pandemic

**DOI:** 10.1002/ajhb.70131

**Published:** 2025-09-17

**Authors:** James K. Gibb, Sarah Williams, Kaspars Mikelstiens, Jada Charles, Leela McKinnon, Laura Beach, Luseadra McKerracher, Jessica Fields

**Affiliations:** ^1^ Department of Anthropology Indiana University Bloomington Indiana USA; ^2^ Kinsey Institute Indiana University Bloomington Indiana USA; ^3^ Department of Anthropology Northwestern University Evanston Illinois USA; ^4^ Department of Health & Society University of Toronto Scarborough Toronto Canada; ^5^ Department of Anthropology University of Connecticut Storrs Connecticut USA; ^6^ Department of Sociology University of British Columbia Vancouver Canada; ^7^ Department of Anthropology University of Toronto Toronto Canada; ^8^ Department of Law & Legal Studies Carleton University Ottawa Canada; ^9^ Department of Public Health Aarhus University Aarhus Denmark; ^10^ Department of Sociology University of Toronto Toronto Canada

**Keywords:** behavioral immune system, food insecurity, LGBT health, perceived infectability, structural equation modeling

## Abstract

**Introduction:**

The COVID‐19 pandemic has disproportionately affected vulnerable populations, including sexual and gender minority (SGM) people. Food insecurity, prevalent among this population, may influence perceived vulnerability to infection and related psychological outcomes. This study investigated the association between food insecurity and perceived vulnerability to infection among SGM adults in Toronto, Canada, during the third wave of the COVID‐19 pandemic.

**Methods:**

A mixed‐methods study was conducted with 338 self‐identified SGM adults recruited via respondent‐driven sampling to complete an internet‐based survey between March and July 2021. Measures included food security status, germ aversion, perceived infectability, and COVID‐19 worry. Structural equation modeling (SEM) examined pathways linking food insecurity, discrimination, sleep quality, and perceived vulnerability to disease, adjusting for demographic and socioeconomic covariates.

**Results:**

The SEM showed that discrimination predicted increased food insecurity (*β* = 0.30, *p* < 0.001) and poorer sleep quality (*β* = 0.26, *p* < 0.001). Sleep quality mediated the relationship between food insecurity and perceived vulnerability to disease (indirect effect = 0.16, *p* < 0.001). Discrimination had a significant total effect on perceived vulnerability to disease (*β* = 0.22, *p* < 0.001).

**Discussion:**

These findings highlight the roles of food insecurity, discrimination, and sleep quality in shaping perceptions of disease vulnerability and risk among SGM people. Interventions addressing food security, mental health, and structural inequities are crucial for mitigating health disparities both during public health crises and in everyday life.

## 1 Introduction

The behavioral immune system (Schaller 2011) refers to any behaviors that protect a person from exposure to viral, bacterial, or fungal threats (Schaller and Park 2011; Shattuck and Muehlenbein 2015); this system operates in concert with a second set of behaviors that promote physiological immune function, generally but not exclusively after an infection has occurred (c.f. Wang et al. 2019). Behaviors that can minimize risks from pathogen exposures can include those related to hygiene and sanitation (e.g., frequent washing of hands and surfaces, boiling of non‐neutral‐smelling water) or those related to avoidance of sources of infection or contagion (e.g., reluctance to touch an infected wound, fear of animal vectors, or even caution around or dislike of unfamiliar people who might be carriers of novel pathogens). Behaviors that can promote immune function, often called “sickness behaviors,” are generally those related to rest or dormancy, as well as those that might signal a need for and elicit care from close others (Wang et al. 2019). These might also include consuming certain foods or medicines that contain the biochemical building blocks necessary for repairing tissues that are damaged or under threat (Wild et al. 2010). Together, the behavioral immune system and the physiological immune promotion system are hypothesized to be underpinned by a suite of evolved cognitive architectures involved in perceiving potential pathogenic threats; assessing their risks and their salience; and motivating a set of responses, typically through disgust, fatigue, anorexia, sadness (to elicit care), and cravings (Schaller and Park 2011; Shattuck and Muehlenbein 2015; Wang et al. 2019).

Life history theory posits that finite energetic and temporal resources are allocated between competing domains of growth, reproduction, and maintenance to shape interspecific biological variation (Stearns 1998). From this perspective, resource allocation toward maintenance represents both physiological processes such as immune function, as well as behavioral processes such as risk perception and pathogen avoidance. Life history theory further suggests that the allocation of resources toward maintenance, including immune function, is influenced by environmental factors such as food security (e.g., Himmelgreen et al. 2022). Thus, a person's perception of vulnerability to disease, an aspect of the behavioral immune system, may be closely tied to that individual's resource availability and stress levels. Furthermore, aspects of the behavioral immune system are activated under different environmental conditions, depending on life history stage, energetic state, and time constraints, as well as the salience and predictability of potential pathogen threats (e.g., Cepon‐Robins et al. 2021). The nutritional environment, particularly with respect to predictability and accessibility, may be an important contributor to behavioral immune responses, given the role of nutrition in energy balance, immune function, and life history tactics as well as the more direct role food plays as a source of pathogens and contaminants (Armelagos 2010, 2014; Barrett et al. 1998; McDade 2005; Valeggia and Ellison 2009).

The evidence regarding the relationship between food insecurity and perception of disease risk to date is sparse, and what is available is ambiguous. Some studies suggest that hunger and nutrition deprivation have no discernible effects on the tendency to avoid pathogen threats or to present signals of pathogen‐avoidant cognition (e.g., Ainsworth and Maner 2014), and other studies suggest that manipulations eliciting experiences of hunger or food unpredictability can evoke responses characteristic of the behavioral immune system (e.g., Perone et al. 2021). Notably, this minimal mixed evidence available to date derives primarily from a cognitively‐, behaviourally‐, and environmentally‐unusual subset of the global population: Western, Educated, Industrialized, Rich, and Democratic (WEIRD) populations, primarily straight, white, college students from North American universities (c.f. Henrich et al. 2010; Clancy and Davis 2019). This subpopulation is relatively unlikely to experience prolonged, severe food insecurity—the sort of condition expected to activate clear behavioral immune responses. Thus, it is critical to integrate evidence on the association between food security and the behavioral immune system in populations outside of WEIRD cohorts.

Food insecurity, defined as the limited or uncertain availability of nutritionally adequate, safe, and culturally appropriate foods or the limited ability to acquire acceptable foods in socially acceptable ways, has been recognized as a significant public health concern in general (Hadley and Crooks 2012) and during the COVID‐19 pandemic in particular (Coleman‐Jensen and Rabbitt 2021). Two‐Spirit, Lesbian, Gay, Bisexual, Transgender, Queer, Questioning, and other non‐heterosexual, non‐cisgender (2SLGBTQ+) peoples, including those living in wealthy, liberal democracies such as Canada and the United States, face structural challenges related to employment, wealth, income, and housing that put them at elevated risk of experiencing food insecurities compared to their heterosexual, cisgender peers (Gibb et al. 2021, 2024; Han and Hernandez 2022; Patterson et al. 2020; Russomanno and Jabson Tree 2020; Sharareh et al. 2023; Testa and Jackson 2021). At the same time, 2SLGBTQ+ people face individual and institutional forms of discrimination which increase psychosocial stress, which has known detrimental impacts on physical health and well‐being (Flentje et al. 2020, 2022) and can be exacerbated by crises such as the COVID‐19 pandemic (Gibb, McKerracher, and Fields 2020). The pandemic has disproportionately impacted marginalized populations, including 2SLGBTQ+ people, possibly leading to increased rates of food insecurity within these communities (Gibb et al. 2024; Hou et al. 2024; Joy 2021; Joy et al. 2025; Singh et al. 2022; Tran et al. 2023).

A growing body of literature has linked food insecurity to adverse health outcomes, including chronic diseases, mental health issues, and, notably, increased vulnerability to infectious diseases (Hadley and Crooks 2012; Gundersen and Ziliak 2015; Weiser et al. 2011; Workman et al. 2021; Himmelgreen et al. 2022). Emergent research on the impacts of adverse nutritional exposures, such as to ultra‐processed foods, which account for at least 50% of the daily caloric intake of severely food insecure households in Canada (Polsky et al. 2024), has demonstrated deleterious impacts on neuropsychiatric health, sleep quality, and immune function (Prescott et al. 2023; Leo et al. 2021).

Sleep plays a critical role in regulating immune function (Faraut et al. 2012), stress response (Martire et al. 2020), and metabolic processes (Cappuccio and Miller 2017), and is therefore an important behavioral pathway through which social and nutritional inequalities may influence health outcomes. Adequate sleep promotes immunity, while chronic sleep disruption has been linked to heightened inflammation and greater susceptibility to disease (Irwin 2012). Sleep disparities are also demonstrated to have important associations with experiences of discrimination (Feagin and Bennefield 2014; Alcántara et al. 2017) and are therefore important to consider in the discussion of social determinants of health and health disparities. These associations are particularly relevant in the context of marginalized populations, such as 2SLGBTQ+ individuals, who are disproportionately exposed to structural and interpersonal forms of discrimination and socioeconomic adversity. Chronic exposure to discrimination can contribute to heightened psychological distress, which in turn disrupts sleep patterns and circadian regulation (See Goosby et al. 2017). Similarly, socioeconomic constraints such as food insecurity, precarious employment, and housing instability may limit one's ability to maintain consistent sleep hygiene and access safe, quiet environments that support rest. Together, these factors help explain the social gradient in sleep quality and position sleep as a key mechanism through which broader social determinants of health shape biological vulnerability and perceived susceptibility to disease.

We might expect, therefore, to find associations between food insecurity and behavioral immune responses related to mitigating vulnerability to infectious diseases, particularly an aspect of behavioral immune cognition called “perceived infectability.” Along with germ aversion–the emotional discomfort arising from contexts in which there may be elevated risk of transmission of pathogens–perceived infectibility is a component of the assessment of vulnerability to disease (Duncan et al. 2009). Perceived infectability specifically refers to a person's subjective assessment of their own susceptibility to infection (Duncan et al. 2009), which affects their patterns of thought and behavior in relation to infectious disease threat (e.g., tendency to believe in or engage in rumors about novel coronavirus transmission Ding and Luo 2022). These perceptions can be influenced by various factors, including salience of endemic and novel pathogen threats in the environment, knowledge of one's own health history, current energy balance and status (or frailty), life‐course stage, predictability of material and immaterial resources, and social network size and dynamics (Murray and Schaller 2016; See also Ding and Luo 2022). To date, however, little is known about intersections between food insecurity and perceived infectability, and the limited data and inferences available concern the WEIRDest subsets of WEIRD samples and populations (i.e., straight, white, college students, c.f. Henrich et al. 2010), to the exclusion of, among others, 2SLGBTQ+ people, their experiences, their cognitions, and their socio‐cultural and economic circumstances.

Thus, the biosocial histories of 2SLGBTQ+ communities within otherwise WEIRD populations may produce experiences and cognitions related to perceived infectability that differ from their non‐2SLGBTQ+ peers that warrant further attention (Gibb, McKerracher, and Fields 2020). The current study aims to examine the association between food insecurity and perceived infectability among a community‐based sample of 2SLGBTQ+ people living in Toronto during the COVID‐19 pandemic, attending to and discussing the biosocial histories of 2SLGBTQ+ communities in Canada for a more nuanced understanding of the relationship between socio‐economic marginalization and immunity in vulnerable populations. Understanding this association may offer insights into the unique health challenges faced by 2SLGBTQ+ people during public health crises and inform future biosocial and evolutionary focused research among sexual and gender diverse populations. Indeed, the COVID‐19 pandemic underscored the need for integrated, biosocial approaches to health that consider not only pathogens but also structural vulnerabilities, social context, and human adaptation (Dimka et al. 2022; van Doren et al. 2024).

Although we cannot measure definitive activation of the behavioral immune system since we lack pre‐COVID samples, we hypothesize that indicators consistent with behavioral immune activation (e.g., heightened perceived vulnerability) will be positively associated with food insecurity. This is because food insecurity is associated with the consumption of ultra‐processed foods, which often contain preservatives and chemicals that promote inflammation, trigger immune‐suppressive mechanisms, and lead to oxidative stress (Lima et al. 2023; Aljahdali et al. 2024; Gowda et al. 2012). At the same time, such foods are deficient in essential micronutrients necessary for cellular maintenance and immune responses. Furthermore, household food insecurity is a form of psychosocial stress, which is known to have biological consequences for cell maintenance and repair (See Walburn et al. 2009). Therefore, people experiencing food insecurity are expected to be more vulnerable to infection, predisposing them to engage in psycho‐social and behavioral immune strategies to compensate for these biological vulnerabilities. We predict a positive association between multiple psychometric indicators of behavioral immune system activation, specifically perceived infectability, germ aversion score, and worry about vulnerability to the novel coronavirus (hereafter, “COVID‐19 worry”).

## 2 Methods

### 2.1 Setting, Design, and Study Populations

This study comprises one set of quantitative analyses on the social epidemiology and health impacts of the COVID‐19 pandemic among 2SLGBTQ+ residents of Toronto, Canada. The survey was embedded in a larger, mixed methods project on the mid‐pandemic health and wellbeing of self‐identified 2SLGBTQ+ adults living in the Greater Toronto Area, Ontario, Canada. These analyses are based on responses to a cross‐sectional, online survey, which was live from March 1, 2021, to July 31, 2021. During this period, COVID‐19 case counts fluctuated slightly but were fairly stable; public health mitigations and restrictions did not change significantly; and overall public health messaging, risk environment, and structural barriers were likewise generally stable. The primary shift during the data collection period was the gradual introduction of the first COVID‐19 vaccines to the population of Ontario, prioritized by category of vulnerability; the elderly and immunocompromised received vaccines first, in the spring months of 2021, and the rest of the population gradually gained access through a variety of mechanisms in the late spring and summer months. However, the proportion of the Toronto population which had received both vaccination doses during the data collection period was extremely small, and given the demographics of our study participants, few were likely to have received even the first dose of the vaccine at the time of their participation. For these reasons, we consider the five‐month data collection period to be fairly stable.

Respondents were recruited using several strategies: (1) a call for participants sent via listservs of organizations serving the Toronto 2SLGBTQ+ communities, and (2) posting to Twitter, Instagram, and Facebook social media platforms. People who expressed interest in participating were invited to complete a pre‐response screener in *Qualtrics* to determine whether they met the inclusion criteria, namely, self‐reported sexual and/or gender minority status and primary residence in the Greater Toronto Area.

Eligible respondents were then invited to complete the 170‐item online survey, also run via *Qualtrics*. The full survey, available in the online Supporting Information (Appendix S1), included multiple modules of items focused on assessing the impact of the ongoing COVID‐19 pandemic on participants' health and wellbeing and included a battery of additional items not reported in this study (measuring psychological, health, behavioral, relational, and social factors). Respondents were compensated for the time they spent on survey completion (an average of ~45–60 min during piloting) with $30 gift cards redeemable with a Canadian grocery chain. This analysis focuses on a sample of 338 participants for whom we have complete data on food security status and their perceived vulnerability to diseases.

### 2.2 Measures of Perceived Vulnerability to Disease

Perceived infectability was assessed using the Perceived Vulnerability to Disease (PVD) questionnaire, a 15‐item scale that measures a person's subjective assessment of their susceptibility to infectious diseases (Duncan et al. 2009). The PVD questionnaire consists of two subscales: perceived infectability (seven items) and germ aversion (eight items). In this study, although we carried out analyses pertaining to both scales, perceived infectability was the primary analytic focus due to its relatively stronger empirical and theoretical relevance, although germ aversion was included in our models as well. Responses were averaged to create a continuous score, with higher scores indicating greater perceived infectability.

COVID‐19‐related worry was evaluated using a 5‐point Likert scale. This scale assessed concerns related to the pandemic, focusing on worries about personal infection, infection of friends or family, and impacts on physical and mental health. Responses ranged from 1 = “Not at all” to 5 = “Extremely,” to rate level of worry for each aspect. Responses were averaged to create a continuous score, with higher scores indicating greater COVID‐19‐related worry.

### 2.3 Food Security Measures

Food security was assessed using the Canadian Household Food Security Survey Module (HFSSM) (Health Canada 2007). The HFSSM consists of 18 questions about financial circumstances affecting food purchasing and consumption, 10 of which focused on adults and eight pertaining to any children (< 18 years of age) living in the respondent's household. Based on the number of affirmative responses, a household's food security was categorized according to one of Canada's four HFI coding thresholds: (1) food secure (no affirmative responses), (2) food insecure, marginal (no more than 1 affirmative response), (3) food insecure, moderate (2–5 affirmative responses), and (4) food insecure, severe (six or more affirmative responses) (c.f., Tarasuk et al. 2013).

### 2.4 Measures of Sexual and Gender Identity

Participants were asked to self‐report their sexual identity/identities, which were categorized into the following groups for the purposes of analyses: (1) Lesbian/Gay, (2) Bisexual/Pansexual, and (3) Other Sexual Identities (OSI) (See Gibb, DuBois, et al. 2020; Gibb et al. 2021; Gibb et al. 2024). Self‐reported gender identity was collapsed into two categories: (1) Transgender and Gender Diverse and (2) Cisgender.

### 2.5 Measures of Household Composition

Household composition was assessed via response to the question: “Do you live alone, with roommates, with a partner, or with family?” Categorical responses included “Alone,” “Roommate,” “Partner,” and “Family.” “Roommate” and “Family” responses included a free response textbox, where respondents were asked to indicate the number of people (excluding themselves) in these categories.

### 2.6 Measures of Sleep Quality

Sleep quality was measured using the Pittsburgh Sleep Quality Index (PSQI) (Buysse et al. 1989), which generates an overall sleep quality score based on 19 self‐reported items with subscales for subjective sleep quality, sleep latency (time it takes to fall asleep), sleep duration, habitual sleep efficiency, sleep disturbances, use of sleeping medication, and daytime dysfunction (e.g., difficulties performing daily activities due to sleepiness). In this analysis, we included the sleep quality and sleep disturbance subscales, with higher scores indicating poorer sleep quality and more frequent sleep disturbances, respectively.

### 2.7 Covariates

Covariates were selected based on their potential to affect perceived vulnerability to disease (germ aversion, perceived infectability, and COVID‐19 worry) and/or the relationships among stressors, household composition, and HFI (Singh et al. 2022; Patterson et al. 2020). These were: age (years) at the time of survey collection; assigned sex at birth (ASAB; male = 1, female = 0); self‐reported race/ethnicity (collapsed into a binary BIPOC status variable to serve as a proxy for respondents' likely experiences with racialization in our statistical models) (See McKerracher et al. 2023), with white participants coded as the reference group in our statistical models; educational attainment (categorized and ranked as: (1) less than 11th Grade, (2) high school/GED, (3) some college or associate degree, and (4) college graduate or higher); and household income (categorized and ranked in Canadian dollar ranges: (1) Under $22 000, (2) $22 000 to $42 000, (3) $42 001 to $62 000, (4) $62 001 to $82 000, (5) $82 001 to $102 000, and (6) over $102 000).

### 2.8 Data Analysis

We report absolute and relative frequencies for categorical variables and mean and standard deviation (SD) for continuous variables. We then performed bivariate analyses to examine the relationships between food security status and key demographic variables, as well as the three main outcome variables: perceived infectability, germ aversion, and COVID‐19 worry. Pearson Chi‐Square tests were used for categorical variables, and independent *t*‐tests were employed for continuous variables to compare the means between food secure and food insecure groups.

To address the complex, multivariate relationships among food security, discrimination, sleep quality, and perceived vulnerability to disease, we also employed structural equation modeling (SEM). SEM allows us to simultaneously test direct and indirect pathways between variables, accommodating both latent constructs and observed variables, and is a recommended approach for studies with designs and variables such as this one (Himmelgreen et al. 2022). We applied SEM here to test a specific, theory‐driven model informed by existing research on health disparities among 2SLGBTQ+ populations (See e.g., Mustanski et al. 2014). This model was designed to examine hypothesized relationships between discrimination, food insecurity, sleep quality, and perceived vulnerability to disease. The model included five latent constructs: Discrimination, Food Security, Sleep Quality, COVID Worry, and Perceived Vulnerability to Disease. Discrimination was measured using the Everyday Discrimination Scale (EDS); Food Security was assessed via the HFSSM (See Section 2.2); Sleep Quality was measured using the PSQI's sleep quality and sleep disturbance subscales; COVID Worry was measured via pandemic‐specific Likert‐scale items; and Perceived Vulnerability to Disease was indicated by the combined scores from the perceived infectability and germ aversion subscales. Covariates included age, assigned sex at birth, racialization (BIPOC status), education, income, sexual orientation, and gender identity.

The SEM incorporated the following key pathways:

- The effect of discrimination on food security and sleep quality;
- The impact of food security and sleep quality on COVID worry; and
- The direct and indirect effects of discrimination, food security, sleep quality, and COVID worry on perceived vulnerability to disease.

Indirect effects were examined to identify mediating pathways, such as the role of sleep quality in the relationship between food insecurity and perceived vulnerability to disease. Model fit was assessed using established indices: Comparative Fit Index (CFI), Tucker‐Lewis Index (TLI), Root Mean Square Error of Approximation (RMSEA), and Standardized Root Mean Square Residual (SRMR). A satisfactory model fit was indicated by CFI ≥ 0.95, TLI ≥ 0.90, RMSEA ≤ 0.06, and SRMR ≤ 0.08. All SEM analyses were conducted using the lavaan package in R (Rosseel 2012).

All analyses were conducted in R (R Core Team 2024), with statistical significance determined at *p* < 0.05.

### 2.9 Ethics Review

This study received ethical approval from the University of Toronto's Social Sciences, Humanities, and Education Research Ethics Board (Protocol #39331).

## 3 Results

### 3.1 Descriptive Statistics

Table 1 presents descriptive statistics for the study sample stratified by food security status. The overall mean age was 27.4 years (SD = 7.40). Most participants were assigned female at birth (AFAB) (70.4%), while 29.6% were assigned male at birth (AMAB). The sample included a diverse racial composition, with 52.4% identifying as BIPOC and 47.6% as white. Our sample had 61.8% of participants identifying as cisgender and 38.2% of participants as transgender or gender diverse (*p* < 0.001). Participants' income levels also varied significantly, with food insecurity most prevalent among those earning under $22 000 CAD (*p* < 0.001). Food insecurity was significantly associated with perceived infectability (*p* = 0.002) and COVID‐19 worry (*p* = 0.026), highlighting disparities in perceived vulnerability to disease. Although germ aversion did not differ by food insecurity status (*p* = 0.166), results suggest its potential variation by other demographic characteristics.

**TABLE 1:** Descriptive statistics for study sample.

	Overall (*N* = 338)	Food secure (*N* = 179)	Food insecure (*N* = 159)	*p*
Age				
Mean (SD)	27.4 (7.40)	27.9 (6.71)	26.7 (8.08)	0.14
Median [min, max]	26.0 [18.0, 63.0]	28.0 [18.0, 56.0]	24.0 [18.0, 63.0]	
Sex assigned at birth				
AMAB	100 (29.6%)	58 (32.4%)	42 (26.4%)	0.278
AFAB	238 (70.4%)	121 (67.6%)	117 (73.6%)	
Racialization				
White	161 (47.6%)	81 (45.3%)	80 (50.3%)	0.412
BIPOC	177 (52.4%)	98 (54.7%)	79 (49.7%)	
Gender identity				
Cisgender	209 (61.8%)	127 (70.9%)	82 (51.6%)	< 0.001
Transgender and gender diverse	129 (38.2%)	52 (29.1%)	77 (48.4%)	
Sexual identity				
Lesbian/gay	139 (41.1%)	85 (47.5%)	54 (34.0%)	0.025
Bisexual/pansexual	132 (39.1%)	59 (33.0%)	73 (45.9%)	
Other sexual identities	67 (19.8%)	35 (19.6%)	32 (20.1%)	
Living situation				
Alone	58 (17.2%)	32 (17.9%)	26 (16.4%)	0.0309
Family	118 (34.9%)	61 (34.1%)	57 (35.8%)	
Partner	86 (25.4%)	55 (30.7%)	31 (19.5%)	
Roommates	76 (22.5%)	31 (17.3%)	45 (28.3%)	
Relationship status				
Married	23 (6.8%)	14 (7.8%)	9 (5.7%)	0.0161
Dating	25 (7.4%)	8 (4.5%)	17 (10.7%)	
In a relationship	134 (39.6%)	80 (44.7%)	54 (34.0%)	
In multiple relationships	14 (4.1%)	3 (1.7%)	11 (6.9%)	
Separated/divorced	4 (1.2%)	3 (1.7%)	1 (0.6%)	
Single	138 (40.8%)	71 (39.7%)	67 (42.1%)	
Education				
College graduate	212 (62.7%)	126 (70.4%)	86 (54.1%)	0.0141
High school/GED	20 (5.9%)	9 (5.0%)	11 (6.9%)	
Less than 11th grade	8 (2.4%)	2 (1.1%)	6 (3.8%)	
Some college	98 (29.0%)	42 (23.5%)	56 (35.2%)	
Income				
Over $102 000	49 (14.5%)	36 (20.1%)	13 (8.2%)	< 0.001
$22 000 to $42 000	69 (20.4%)	25 (14.0%)	44 (27.7%)	
$42 001 to $62 000	62 (18.3%)	32 (17.9%)	30 (18.9%)	
$62 001 to $82 000	35 (10.4%)	21 (11.7%)	14 (8.8%)	
$82 001 to $102 000	33 (9.8%)	26 (14.5%)	7 (4.4%)	
Under $22 000	90 (26.6%)	39 (21.8%)	51 (32.1%)	
Perceived vulnerability to disease				
Mean (SD)	3.99 (0.919)	3.86 (0.861)	4.13 (0.962)	0.007
Median [min, max]	4.00 [1.33, 6.67]	3.80 [1.60, 6.47]	4.13 [1.33, 6.67]	
Germ aversion score				
Mean (SD)	4.18 (1.00)	4.11 (1.01)	4.26 (0.985)	0.165
Median [Min, Max]	4.13 [1.63, 6.88]	4.13 [1.88, 6.63]	4.25 [1.63, 6.88]	
Perceived infectability score				
Mean (SD)	3.77 (1.21)	3.58 (1.09)	3.98 (1.30)	0.002
Median [min, max]	3.71 [1.00, 7.00]	3.43 [1.00, 6.57]	3.86 [1.00, 7.00]	
Everyday discrimination scale score				
Mean (SD)	22.5 (8.25)	19.7 (7.08)	25.6 (8.37)	< 0.001
Median [min, max]	21.0 [9.00, 52.0]	19.0 [9.00, 51.0]	24.0 [9.00, 52.0]	
COVID‐19 worry score				
Mean (SD)	15.8 (3.72)	15.4 (3.79)	16.3 (3.59)	0.026
Median [min, max]	16.0 [4.00, 25.0]	16.0 [4.00, 25.0]	16.0 [6.00, 25.0]	
Food security status				
Secure	179 (53.0%)	179 (100%)	0 (0%)	< 0.001
Marginal	69 (20.4%)	0 (0%)	69 (43.4%)	
Moderate	48 (14.2%)	0 (0%)	48 (30.2%)	
Severe	42 (12.4%)	0 (0%)	42 (26.4%)	
HIV status				
Negative	290 (85.8%)	160 (89.4%)	130 (81.8%)	0.078
Positive	17 (5.0%)	5 (2.8%)	12 (7.5%)	
Unknown	31 (9.2%)	14 (7.8%)	17 (10.7%)	
How much are you reading or talking about COVID‐19?				
Never/rarely	26 (7.7%)	13 (7.3%)	13 (8.2%)	0.157
Most of the time	52 (15.4%)	35 (19.6%)	17 (10.7%)	
Occasionally	104 (30.8%)	51 (28.5%)	53 (33.3%)	
Often	156 (46.2%)	80 (44.7%)	76 (47.8%)	
Sleep quality score				
Mean (SD)	1.39 (0.686)	1.28 (0.620)	1.51 (0.738)	0.003
Median [min, max]	1.00 [0, 3.00]	1.00 [0, 3.00]	1.50 [0, 3.00]	
Missing	1 (0.3%)	0 (0%)	1 (0.6%)	
Sleep disturbance score				
Mean (SD)	1.42 (0.587)	1.30 (0.528)	1.55 (0.623)	< 0.001
Median [min, max]	1.00 [0, 3.00]	1.00 [0, 3.00]	2.00 [0, 3.00]	

### 3.2 Structural Equation Models

**FIGURE 1:**
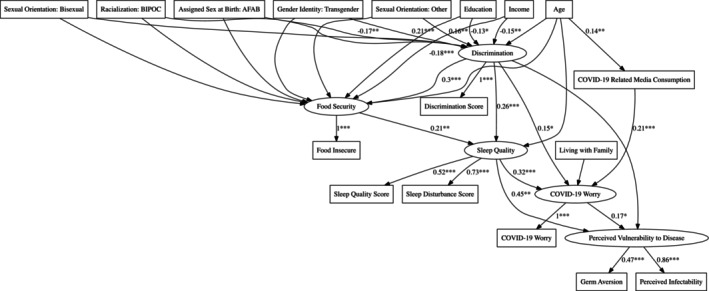
Structural equation model plot. *p < 0.05; **p < 0.01; ***p < 0.001.

The SEM (Figure 1; Tables 2, 3, 4, 5) revealed pathways linking discrimination, food insecurity, sleep quality, and perceived vulnerability to disease. Discrimination significantly predicted food insecurity (*β* = 0.30, SE = 0.05, *p* < 0.001) and poorer sleep quality (*β* = 0.29, SE = 0.06, *p* < 0.001). Food insecurity further contributed to sleep disturbances (*β* = 0.19, SE = 0.06, *p* < 0.01). Sleep quality, in turn, mediated the relationship between food insecurity and perceived vulnerability to disease (indirect effect = 0.16, *p* < 0.001). COVID‐19 worry (*β* = 0.21, SE = 0.06, *p* < 0.01), where food insecurity is associated with poorer sleep quality, which in turn is associated with greater perceived vulnerability to disease. Sleep quality (*β* = 0.40, SE = 0.08, *p* < 0.001) significantly influenced perceived vulnerability to disease. Notably, indirect pathways revealed cumulative impacts of discrimination and food insecurity mediated through sleep quality and COVID‐19 worry. The total effect of discrimination on perceived vulnerability to disease was significant (*β* = 0.22, SE = 0.06, *p* < 0.001). Fit indices supported the model's adequacy (CFI = 0.95, TLI = 0.92, RMSEA = 0.03, SRMR = 0.04). Variance explained included food security (*R* ^2^ = 0.20), sleep quality (*R* ^2^ = 0.16), and perceived vulnerability to disease (*R* ^2^ = 0.32).

**TABLE 2:** Structural equation model fit and significance.

N	*χ* ^2^	df	*p*	CFI	RMSEA	90% CI	TLI	SRMR	AIC	BIC
337	88.42	68	0.05	0.95	0.03	0.01–0.05	0.92	0.04	8544.47	8697.27

**TABLE 3:** Factor loadings.

Latent factor	Indicator	Standardized				
		Loading	95% CI	SE	*z*	*p*
COVID‐19 worry	COVID‐19 worry score	1.00	1.00–1.00	0.00		
Discrimination	Everyday discrimination score	1.00	1.00–1.00	0.00		
Food security	Food insecure	1.00	1.00–1.00	0.00		
Perceived vulnerability to disease	Germ aversion score	0.47	0.35–0.60	0.06	7.50	< 0.001
Perceived vulnerability to disease	Perceived infectability score	0.86	0.69–1.03	0.09	9.96	0.00
Sleep quality	Sleep quality score	0.73	0.60–0.86	0.06	11.38	0.00
Sleep quality	Sleep disturbance score	0.52	0.40–0.63	0.06	8.81	0.00

* *p* < 0.05.

** *p* < 0.01.

*** *p* < 0.001.

**TABLE 4:** Structural equation model regression paths, direct, indirect, and total effects.

Predictor	DV	Label/pathway	Standardized				
			*β*	95% CI	SE	*z*	*p*
COVID‐19 related media consumption	COVID‐19 worry		0.21	0.11–0.30	0.05	4.22	< 0.001
Discrimination	COVID‐19 worry	c	0.15	0.04–0.26	0.06	2.58	0.01
Living with family (ref. Living alone)	COVID‐19 worry		0.07	−0.03–0.16	0.05	1.34	0.18
Sleep quality	COVID‐19 worry	b	0.32	0.19–0.46	0.07	4.65	< 0.001
Age	COVID‐19 related media consumption		0.14	0.04–0.25	0.05	2.68	0.01
Age	Discrimination		0.05	−0.06–0.16	0.06	0.91	0.36
Assigned female at birth	Discrimination		−0.17	−0.28–−0.06	0.06	−2.93	0.00
Education	Discrimination		−0.13	−0.23–−0.02	0.05	−2.36	0.02
Transgender/gender diverse (ref. Cisgender)	Discrimination		0.21	0.10–0.31	0.05	3.92	< 0.001
Income	Discrimination		−0.15	−0.26–−0.05	0.05	−2.98	0.00
BIPOC (ref. White)	Discrimination		0.08	−0.02–0.18	0.05	1.49	0.14
Bisexual (ref. lesbian/gay)	Discrimination		0.08	−0.04–0.20	0.06	1.37	0.17
Other sexual identities	Discrimination		0.16	0.05–0.28	0.06	2.73	0.01
Age	Food security		0.01	−0.09–0.12	0.06	0.24	0.81
Assigned female at birth	Food security		0.01	−0.10–0.12	0.06	0.23	0.82
Discrimination	Food security	g	0.30	0.20–0.40	0.05	6.02	< 0.001
Education	Food security		−0.10	−0.20–0.01	0.05	−1.82	0.07
Transgender/gender diverse (ref. Cisgender)	Food security		0.10	0.00–0.20	0.05	1.88	0.06
Income	Food security		−0.18	−0.28–−0.08	0.05	−3.57	< 0.001
BIPOC (ref. White)	Food security		−0.10	−0.19–0.00	0.05	−1.97	0.05
Bisexual (ref. Lesbian/gay)	Food security		0.07	−0.05–0.18	0.06	1.10	0.27
Other sexual identities	Food security		−0.04	−0.15–0.07	0.06	−0.67	0.50
COVID‐19 worry	Perceived vulnerability to disease	e	0.17	0.03–0.30	0.07	2.45	0.01
Discrimination	Perceived vulnerability to disease	f	0.05	−0.08–0.18	0.07	0.81	0.42
Sleep quality	Perceived vulnerability to disease	d	0.45	0.27–0.64	0.09	4.84	< 0.001
Age	Sleep quality		0.09	−0.03–0.22	0.06	1.45	0.15
Discrimination	Sleep quality	a	0.26	0.12–0.40	0.07	3.65	< 0.001
Food security	Sleep quality	h	0.21	0.08–0.34	0.07	3.11	0.00
a*d	ind1	Discrimination → sleep quality → perceived vulnerability to disease	0.12	0.03–0.20	0.04	2.73	0.01
a*b*e	ind2	Discrimination → sleep quality → COVID‐19 worry → perceived vulnerability to disease	0.01	0.00–0.03	0.01	2.03	0.04
g*h*d	ind3	Discrimination → food security → sleep quality → perceived vulnerability to disease	0.03	0.01–0.05	0.01	2.37	0.02
g*h*b*e	ind4	Discrimination → food security → sleep quality →COVID‐19 worry → perceived vulnerability to disease	0.00	0.00–0.01	0.00	1.83	0.07
ind1 + ind2 + ind3 + ind4	Indirect	The total indirect effect from discrimination to perceived vulnerability to disease	0.16	0.07–0.26	0.05	3.41	< 0.001
F + Indirect	Total	Total effect of discrimination on perceived vulnerability to disease	0.22	0.09–0.34	0.06	3.47	< 0.001

* *p* < 0.05.

** *p* < 0.01.

*** *p* < 0.001.

**TABLE 5:** Explained variance.

Variable	*R* ^2^
Food security	0.20
Sleep quality	0.16
Discrimination	0.14
COVID‐19 worry	0.20
Perceived vulnerability to disease	0.32

## 4 Discussion

### 4.1 Main Findings

Our study explored the association between food insecurity and perceived infectability among a community‐based sample of 2SLGBTQ+ people living in Toronto during the COVID‐19 pandemic. We found that food insecurity was significantly associated with higher perceived infectability scores, even after adjusting for multiple demographic, psychological, and socioeconomic covariates. This suggests that food‐insecure people may feel more vulnerable to infections, potentially leading to compensatory behaviors linked to the behavioral immune system, such as increased hygiene practices, risk avoidance, and heightened vigilance in health‐related contexts. In addition to food insecurity, age was a consistent predictor across all three outcome measures: germ aversion, perceived infectability, and COVID‐19 worry, indicating that older people might perceive themselves as more vulnerable to infections. These perceptions are likely influenced by media and public health messages that emphasize the increased vulnerability of older populations (Delporte et al. 2023), as well as greater sensitivity to and attention paid to personal health that accompany the transition out of adolescence and emerging adulthood, particularly as individuals begin to take on adult roles and plan for their future (McDade et al. 2011). Moreover, beyond age and standardized metrics of food insecurity, our findings highlight the need to consider social factors like living arrangements, perceived social support, and everyday discrimination when examining the experiences of 2SLGBTQ+ people during a pandemic, as these factors may contribute to perceived vulnerability and associated psychological distress.

Our findings partially support our hypotheses regarding the relationship between food insecurity and indicators of behavioral immune activation. As hypothesized, food insecurity was positively associated with perceived infectability, suggesting that people experiencing food insecurity may indeed feel more vulnerable to infection. This aligns with the notion that food insecurity, which is often linked to the consumption of ultra‐processed foods and associated nutritional deficiencies, and which is consistently linked with increased psycho‐social stress, could heighten immune‐suppressive mechanisms and oxidative stress (Lima et al. 2023; Aljahdali et al. 2024; Gowda et al. 2012; Bermúdez‐Millán et al. 2019). We speculate that these biological vulnerabilities trigger compensatory psycho‐social and behavioral immune strategies, such as increased perceived susceptibility to illness. Further research is needed to explore these dynamics and to understand the specific conditions under which food insecurity influences different aspects of behavioral immune activation.

### 4.2 Biosocial Factors Shaping the Behavioral Immune System

Our findings reveal important relationships between food insecurity, discrimination, sleep quality, and perceived vulnerability to disease. Notably, our analyses highlight that the effects of discrimination and food insecurity are mediated through sleep quality. We found that food insecurity is associated with poor sleep quality, and that poor sleep quality in turn is linked to greater perceived vulnerability to disease. These results, along with other recent studies, support sleep's important role in linking stressors such as perceived discrimination to poor health outcomes (Slopen et al. 2016). In our analyses, higher sleep quality is significantly associated with lower perceived vulnerability to disease and mediates the relationship between food insecurity and disease vulnerability. Stressors like discrimination and food insecurity are thought to elicit feelings of threat, leading to increased physiological and psychological arousal, which in turn disrupts sleep quality (Harrell et al. 2003; Troxel et al. 2020). Our results further support the idea that sleep disruption may amplify feelings of risk, and demonstrate the bidirectional link between sleep quality and perceived disease vulnerability to identify multiple pathways through which food insecurity may activate behavioral immune responses.

This study has important implications for understanding human biology and biosocial variation of behavioral immune responses in populations that are less represented in WEIRD samples. Our findings emphasize the critical role of food insecurity as a factor shaping behavioral immune responses among 2SLGBTQ+ people facing disease threats like COVID‐19. High levels of food insecurity, relatively prevalent among non‐WEIRD or less WEIRD samples, involve ongoing unpredictability of essential resources and related stress, which likely increase the salience of infectious disease threats and influence how people perceive and respond to these threats (Hadley and Patil 2008; Hadley and Crooks 2012). Addressing food insecurity in marginalized populations, such as 2SLGBTQ+ communities, should be a priority for evolutionary medicine, as doing so would not only improve overall health through better nutrition and reduced stress but also would likely positively influence behavioral immune responses, which can drive social isolation and distrust (Schaller 2011; Schaller et al. 2015).

The study's findings can be interpreted through the twin lenses of the anticipatory mechanisms of the behavioral immune system, which operate to minimize infection risk, and of peri‐ or post‐infection mechanisms of physiological immune system promotion, including sickness behaviors and food/medicine consumption. Together, these two apparatuses are adaptive mechanisms evolved to respond to environmental stressors like infection risk and food insecurity. Sickness behavior includes behavioral changes such as increased sleep, reduced activity, social withdrawal, and loss of appetite, which all conserve energy and facilitate recovery (Hart 1988). In this context, food‐insecure people may exhibit a pathological form of sickness behavior in the absence of infection due to the chronic stress of their situation, with factors like social withdrawal and reduced activity ultimately exacerbating their vulnerability to infections. On the other hand, the heightened COVID‐19 worry among food‐insecure participants may reflect an adaptive increase in vigilance to protect against perceived threats. The behavioral immune system, which is argued to mitigate infection risk by motivating the avoidance of potential pathogens (Schaller and Park 2011) appears to be activated in food‐insecure people in this LGBTQ+ sample from Toronto, as indicated by their higher perceived infectability. We might also expect these food‐insecure individuals to be relatively motivated to engage in immune‐promoting sickness behaviors or eating/medicine‐taking behaviors (c.f. Fessler 2002; McKerracher et al. 2016) but unfortunately, these data were not collected, so we can only speculate. Regardless, these increased perceived infectability responses may be adaptive, as people whose immune systems are compromised due to inadequate nutrition would benefit from minimizing pathogen risks.

### 4.3 Public Health and Social Policy Implications

The high prevalence of food insecurity among 2SLGBTQ+ adults in our study underscores the urgent need for targeted interventions to address the social determinants of health in this population. Public health policies should prioritize alleviating food insecurity, particularly during crises like the COVID‐19 pandemic, by addressing root causes such as poverty, housing instability, and employment discrimination (Gibb et al. 2024). Such efforts could improve the overall health and well‐being of 2SLGBTQ+ people and reduce their vulnerability to infectious diseases. Our findings also highlight the need for interventions that address both the physical and psychological aspects of food insecurity. These interventions might include ensuring access to nutritious food, providing resources for managing stress and promoting mental health, and fostering social support networks to mitigate the negative consequences of food insecurity on immune function and well‐being. It is particularly important to consider the unique challenges faced by transgender and gender diverse people, who reported higher perceived infectability scores and may face additional barriers to accessing healthcare, nutritious food, and social support.

### 4.4 Limitations and Strengths

This study has several limitations. The cross‐sectional design limits our ability to infer causality between food insecurity and perceived infectability, and longitudinal studies are needed to explore these relationships over time. Additionally, the reliance on self‐report measures introduces the potential for social desirability and recall biases. Future research should, to the extent possible, incorporate objective measures of food insecurity and immune functioning to provide a more comprehensive understanding of these associations among 2SLGBTQ+ people. More careful modeling of all outcomes (including germ aversion which we chose not to center in the present analyses) as functions of multiple potential social and demographic covariates would also strengthen our understanding of these associations. Moreover, the generalizability of our findings may be limited to the specific sample of 2SLGBTQ+ adults living in Toronto, and further research is needed to explore these associations in other populations and geographic locations. However, incorporating 2SLGBTQ+ perspectives into evolutionary medicine and biological anthropology research is essential for advancing our understanding of human biology and health. Including these perspectives challenges traditional assumptions derived from predominantly heterosexual and cisgender WEIRD populations and promotes more inclusive and equitable research approaches.

Despite the limitations, the study has several notable strengths, including our use of SEM, which allowed for a comprehensive exploration of the biosocial pathways shaping the relationship between food insecurity and perceived vulnerability to infection among 2SLGBTQ+ adults. This integrative approach allowed us to test complex, multivariate relationships while quantifying both direct and mediated effects among study variables. By leveraging SEM, we enhanced our ability to capture the interplay between biosocial factors shaping perceptions of risk during the COVID‐19 pandemic. Future research should prioritize longitudinal studies to establish causal links between food insecurity, psychological distress, and changes in perceived and physiological vulnerability to infection, particularly in 2SLGBTQ+ populations. Additionally, developing and validating targeted public health interventions to address food insecurity and its associated health outcomes in these vulnerable communities should be a priority to improve health equity and reduce health disparities (Gibb et al. 2024; Joy 2021; Joy et al. 2025).

## 5 Conclusion

In conclusion, this study offers insight into the association between food insecurity and perceived infectability among a community‐based sample of 2SLGBTQ+ people living in Toronto during the COVID‐19 pandemic. The results of our study suggest that food insecurity may contribute to heightened perceived vulnerability to infection in this population, which could have implications for their overall health and well‐being during public health crises. The findings reveal a significant positive association between food insecurity and perceived infectability, even after adjusting for multiple demographic, socioeconomic, and psychosocial factors. These results contribute to a growing body of literature on the impact of food insecurity on health and well‐being and highlight the importance of addressing this issue among 2SLGBTQ+ populations.

By incorporating perspectives from the evolutionary, social, and behavioral health sciences, researchers can better understand the complex interplay between food security, psychosocial factors, and health among 2SLGBTQ+ people. These perspectives shed light on the adaptive mechanisms that have evolved in response to environmental stressors and inform public health interventions aimed at alleviating food insecurity and its consequences. The study underscores the need for targeted interventions that address the unique challenges faced by 2SLGBTQ+ people, such as minority stress, and improve access to nutritionally adequate and safe foods to promote overall health and well‐being. Future research should address these limitations by employing longitudinal designs, incorporating objective measures of food insecurity and immune functioning, and exploring the association between food insecurity and perceived infectability in diverse populations and geographic locations.

Considering the COVID‐19 pandemic and the potential for future public health crises, it is crucial to address the multifaceted challenges faced by food‐insecure populations, particularly among vulnerable groups such as 2SLGBTQ+ people. By understanding the complex relationships between food security, psychosocial factors, and health through the lenses of evolutionary medicine, we can inform effective public health interventions and promote health equity for all.

## Conflicts of Interest

The authors declare no conflicts of interest.

## Supporting information


**Data S1:** Supporting Information.

## Data Availability

The data that support the findings of this study are available on request from the corresponding author. The data are not publicly available due to privacy or ethical restrictions.
