# Supplementary material for: Biosocial Factors Shaping Perceptions of Disease Risk Among a Community‐Based Sample of Sexual and Gender Minority People Living in Toronto During the COVID‐19 Pandemic
Source: Am J Hum Biol. 2025 Sep 17;37(9):e70131. doi: 10.1002/ajhb.70131 (PMC12441999; doi:10.1002/ajhb.70131)
Supplement: Supplementary file 1 — Data S1: Supporting Information. [file AJHB-37-e70131-s001.docx]

Queers & COVID-19 4.0

Start of Block: Captcha & Screening

Q1.1

Q1.2 Click to write the question text

Browser (1)

Version (2)

Operating System (3)

Screen Resolution (4)

Flash Version (5)

Java Support (6)

User Agent (7)

End of Block: Captcha & Screening

Start of Block: Introduction

Q2.1 **Consent for Research Participation**   **Title of Study:** Impact of COVID-19 on the Mental Health and Vulnerability of Sexual and Gender Minorities Living in Toronto    **Researcher(s):**                                                             Dr. Jessica Fields, Principal InvestigatorJames K Gibb, Project Manager
 **Email:** queercovid.utsc@utoronto.ca      You are invited to take part in a study to assess how the COVID-19 pandemic and associated public health measures aimed at controlling COVID-19 transmissions (e.g. social distancing, self-isolation) may impact the mental health of sexual and gender minorities (SGMs) living in Toronto. The box below highlights key information about this research for you to consider when making a decision whether or not to participate. Carefully consider this information and the more detailed information provided below the box. Please ask questions about any of the information you do not understand before you decide whether to participate.   **Key Information for You to Consider** **Voluntary Consent**: You are being asked to volunteer for a research study. It is up to you whether you choose to participate or not. There will be no penalty or loss of benefits to which you are otherwise entitled if you choose not to participate or discontinue participation.    **Purpose:** The aims of this research are to document and assess: 1) how COVID-19 is influencing the mental health and well-being of SGMs; 2) how SGMs cope with and respond to current COVID-19 control measures; 3) the sources of SGMs’ COVID-19 knowledge; 4) the socioeconomic factors shaping how SGMs experience during COVID-19 crisis; 5) bring to light any concerns that SGMs may have regarding COVID-19.   **Duration:** It is expected that your participation will last 45-50 minutes during  survey.    **Procedures and Activities:**You will be asked to participate in an online survey on COVID-19. The survey will ask you some questions about yourself, such as your age, gender identity, sexual orientation, socioeconomic status, as well as some questions about your mental health, sleep,  behaviours and experiences during the COVID-19 pandemic. The survey will contain questions about your mental health and behaviours during COVID-19 pandemic, your experiences during COVID-19, and perceptions of your own risk of COVID-19.   **Potential Risks:**It is not likely that there will be any harms or discomforts associated with participating in this study, though since you are discussing your mental health and how COVID-19 has influenced your experiences over the past couple months you may feel anxious or upset. A list of mental health resources will be available during and after the survey if you would like more information. You can stop taking part in the study at any time.    **Potential Benefits:**The research may not benefit you directly, but it will give the opportunity for participants to receive and engage with information regarding their health in a non-clinical and, ideally, supportive setting. Participating in this project will provide invaluable information on how SGMs are impacted by COVID-19, enabling Toronto and other Canadian public health agencies to better respond to the needs of SGMs and other vulnerable communities during this and future health crises. This project will also raise awareness on how current sociopolitical and public health crises impact the community health of SGM people(s) living in Toronto.    **Confidentiality:** All information collected as part of this research project will remain confidential within the limits provided by the law. You are participating in this study confidentially. We will not use your name or any information that would allow you to be identified in any publications or presentations. All participants will be assigned an ID number, rather than your real name. Jessica Fields, PI, and James K Gibb, Project Manager, will be the only persons with access to the raw data for this project. Electronic files (notes) will be password-protected and encrypted on Dr. Fields’ and Mr. Gibbs’ computers. During COVID-19 physical distancing, physical files (notes) will be kept in a locked file cabinet in Dr. Fields’ home office. When public health measures allow the team to return to the University of Toronto campus, physical files will be stored in Dr. Fields’ office. Study ID numbers and descriptions will be kept separate from data. Access to digital files will be password protected, and data will only be transferred on password protected USB sticks. Keys for field notes and written transcripts will be in Dr. Fields’ and Mr. Gibbs’ possession only. No other people beyond members of the study team will have access to this data. No data will be uploaded to a central "cloud"-type server. Once the study is complete the research data, including audio files and paper copies of surveys, will be maintained for five years and then destroyed via file deletion.    **Participation and Withdrawal:**You may withdraw your consent to participate in any portion of the study at any time by contacting the PI until data analysis begins in June 2021. At that time, results will be distributed, and consent can no longer be withdrawn.   **Compensation:**Upon completion of the survey you will receive a $30.00 gift card. If you choose to withdraw prior to completing the survey, you will receive a gift card in the amount equal to the percentage of survey you completed (e.g. 50% = $15.00).   **Information about the Study:**We expect to have preliminary results by June of 2021. If you are interested in receiving a summary of results or any material produced from this research please contact Dr. Jessica Fields or James Gibb.    This study has been reviewed by the Social Sciences, Humanities and Education Research Ethics Board at the University of Toronto and received ethics clearance (RIS Protocol Number 00039331).   **Questions:** Jessica Fields, PhD, Principal Investigator James K Gibb, Project Manager
 **Email:** queercovid.utsc@utoronto.ca                 For questions about your rights as a research participant, you may contact The University of Toronto’s Office of Research Oversight and Compliance at 416-946-3273, or at ethics.review@utoronto.ca     **Do you consent to these terms?**Clicking on the “Yes” button below will start the survey and confirms your consent to participate in this study.

- Yes (1)
- No (2)

Skip To: End of Survey If Consent for Research Participation   Title of Study: Impact of COVID-19 on the Mental Health and... != Yes

End of Block: Introduction

Start of Block: Additional Study Opportunities

Q3.1 Would you be interested in being contacted again in the future phases of this project (e.g. interviews, focus groups, follow-up health surveys)?

- Yes (4)
- No (5)

Q3.2 How would you like to receive your remuneration for participating in our study?

- Door Dash Gift Card (1)
- PayPal (4)
- President's Choice Gift Card (6)
- Prefer not to be remunerated (5)

Q3.3 To receive your remuneration for participating, please write your email in the box below. Providing your email does NOT mean you have consented to participate or that you are obligated to agree to participate in future phases of this project. Please allow 2 weeks for your compensation to be sent. If choosing option 3, please note that you must have a verified Canadian PayPal account in order to accept a PayPal money transfer. Please provide the email address associated with your PayPal account.

________________________________________________________________

End of Block: Additional Study Opportunities

Start of Block: Socio-Demographics

Q4.1 What is your age, in years?

________________________________________________________________

Q4.2 What sex were you assigned at birth?

- Male (1)
- Female (2)
- Undetermined (3)
- Other. Specify: (4) ________________________________________________

Q4.3 Which of the following best describes your gender identity?

- Agender (1)
- Androgyne (2)
- Bigender (3)
- Cisgender man/ Cis man (4)
- Cisgender woman/Cis women (5)
- Genderfluid (6)
- Genderqueer (7)
- Non-binary (8)
- Transgender woman/Trans woman (9)
- Transgender man/Trans man (10)
- Two Spirit (12)
- Other. Specify: (11) ________________________________________________

Q4.4 If you had to select one response that best describes your current gender for the purposes of a survey, what would it be?

- Man or boy (1)
- Woman or girl (2)
- Indigenous or other cultural identity (e.g. two-spirit) (4)
- Non-binary, genderqueer, agender or a similar identity (5)
- Other. Specify: (3) ________________________________________________

Q4.5 What best describes the gender you currently live in your day-to-day life?

________________________________________________________________

Q4.6 Which of these commonly used sexual orientation categories best captures your identity? Check all that apply.

- Asexual (1)
- Bisexual (2)
- Gay (3)
- Heterosexual/straight (4)
- Lesbian (5)
- Pansexual (6)
- Queer (7)
- Other. Specify: (8) ________________________________________________

Q4.7 What best describes your race or ethnicity? Select all that apply.

- Arab (1)
- Asian, East (e.g. Chinese, Japanese, Korean) (2)
- Asian, South (e.g., East Indian, Pakistani, Sri Lankan) (3)
- Asian, Southeast (e.g., Vietnamese, Cambodian, Malaysian, Laotian) (4)
- Asian, West (e.g., Iranian, Afghan) (5)
- Black (6)
- Filipino (7)
- Indigenous (e.g. First Nations, Métis or Inuk (Inuit), Status and Non-Status Indian)? (12)
- Latin American (8)
- Mixed (9)
- White (10)
- Other. Specify: (11) ________________________________________________

Display This Question:

If What best describes your race or ethnicity? Select all that apply.  = Indigenous (e.g. First Nations, Métis or Inuk (Inuit), Status and Non-Status Indian)?

Q4.8 Are you Inuk (Inuit), First Nations, or Métis?

- Inuk (Inuit) (1)
- First Nations (4)
- Métis (5)

Q4.9  Were you born in Canada?

- Yes (1)
- No (2)

Skip To: Q4.11 If  Were you born in Canada? = Yes

Q4.10 If you were born outside of Canada, how many years have you lived in Canada?

- Less than one year (1)
- 1-5 years (4)
- 5-10 years (2)
- more than 10 years (3)

Q4.11 Are you currently employed, either full or part time?

- No (1)
- Yes, I am self-employed (2)
- Yes, I am employed by others part time (less than 35 hours per week) (3)
- Yes, I am employed by others full-time (35 or more hours per week) (4)

Q4.12 Please tell us your current occupation.

________________________________________________________________

Q4.13 What is the highest degree or level of school you have completed? If you are currently enrolled, please indicate the highest level or degree you have attained so far.

- None (1)
- Primary, elementary and/or junior high (2)
- Some high school (3)
- High school graduate – high school diploma or equivalent (i.e. GED) (4)
- Some college/university credits, but less than 1 year (5)
- Technical or vocational school degree or diploma (6)
- One or more years of college/university, no degree (7)
- Associate’s degree or college diploma (8)
- Bachelor’s degree (9)
- Master’s degree (10)
- Doctorate or professional degree (e.g. PhD, MD, JD, DDS) (11)
- Graduate of a Certificate Program (12)

Q4.14 Are you currently pregnant?

- Yes (1)
- No (2)
- Not sure (3)

Q4.15  What is your current relationship status? Check all that apply.

- Single (1)
- Dating (2)
- In a relationship (3)
- In multiple relationships (4)
- Married (5)
- Separated/Divorced (6)

Skip To: Q4.17 If  What is your current relationship status? Check all that apply. = Single

Q4.16 How would you describe this/these relationships? Some people might be in a monogamous relationship, polyamorous relationship, or open relationship, etc.

________________________________________________________________

Q4.17 Do you live alone, with roommates, with a partner, or with family?

- Alone (1)
- Roommate (how many roommates, excluding yourself?) (2) ________________________________________________
- Partner (3)
- Family (how many family members, excluding yourself?) (4) ________________________________________________

Q4.18 Please indicate your income or your household income before taxes in the last year, whichever is greater. If you do not earn an income, please select N/A.

- N/A (1)
- Under $22,000 (2)
- $22,000 to $42,000 (3)
- $42,000 to $62,000 (4)
- $62,000 to $82,000 (5)
- $82,000 to $102,000 (6)
- over $102,000 (7)

Q4.19 Have you ever been homeless?

- Yes (1)
- No (2)

Skip To: Q4.22 If Have you ever been homeless? = No

Q4.20 At what age did you experience being homeless?

________________________________________________________________

Q4.21 Have you experienced homelessness more than once in your life?

- Yes (1)
- No (2)

Q4.22 Is there anyone living in your home who is employed as an essential worker (e.g., healthcare, delivery worker, store worker, security, building maintenance)?

- Yes (3)
- No (2)

Skip To: End of Block If Is there anyone living in your home who is employed as an essential worker (e.g., healthcare, del... = No

Q4.23 Do they come home each day?

- Yes (1)
- No, they stay away from home due to COVID-19 (2)
- No, they stay away from home for other reasons (3)

Q4.24 Are any members of the household a first responder, healthcare provider, or other worker in a facility treating COVID-19?

- Yes (1)
- No (2)

End of Block: Socio-Demographics

Start of Block: Sex & Gender

Q5.1 Have you ever had sex ?

- Yes (32)
- No (34)
- Prefer not to say (35)

Skip To: Q5.6 If Have you ever had sex ? = No

Skip To: Q5.6 If Have you ever had sex ? = Prefer not to say

Q5.2 **In your lifetime**, how many **casual**sex partners have you had?

________________________________________________________________

Q5.3 In your lifetime, how many of your casual sex partners were… (Please enter a number next to each option -  enter “0” if you have not had a partner of that gender)

- Cisgender men/Cis men (persons who were assigned male at birth and identify as a man): (1) ________________________________________________
- Transgender man/Trans man (persons who were assigned female at birth and now identify as a man, FTM): (4) ________________________________________________
- Cisgender women/Cis women (persons who were assigned female at birth and identify as women): (5) ________________________________________________
- Transgender women /Trans women (persons who were assigned male at birth and now identify as women, MTF): (6) ________________________________________________
- Non-binary, Genderqueers, or other genders: (7) ________________________________________________
- Two Spirit, or other culturally specific genders: (8) ________________________________________________

Q5.4 In the past 12 months, how many casual sex partners have you had?

________________________________________________________________

Q5.5 In the past 12 months, how many of your casual sex partners were… (Please enter a number next to each option -  enter “0” if you have not had a partner of that gender)

- Cisgender men/Cis men (persons who were assigned male at birth and identify as a man): (1) ________________________________________________
- Transgender man/Trans man (persons who were assigned female at birth and now identify as a man, FTM): (4) ________________________________________________
- Cisgender women/Cis women (persons who were assigned female at birth and identify as women): (5) ________________________________________________
- Transgender women /Trans women (persons who were assigned male at birth and now identify as women, MTF): (6) ________________________________________________
- Non-binary, Genderqueers, or other genders: (7) ________________________________________________
- Two Spirit, or other culturally specific genders: (8) ________________________________________________

Q5.6 Please indicate how often you have engaged in the following sexual behaviors during the COVID-19 pandemic:

|  | Never (1) | Few times during the pandemic (3) | Few times a month (4) | Once a week (5) | Few times a week (6) | Daily (7) |
| --- | --- | --- | --- | --- | --- | --- |
| Kissing (1) |  |  |  |  |  |  |
| French Kissing (2) |  |  |  |  |  |  |
| Touching a partner's chest or having your chest touched by a partner (3) |  |  |  |  |  |  |
| Stimulating a partner's genitals or having your genitals stimulated by a partner (4) |  |  |  |  |  |  |
| Performing oral sex (6) |  |  |  |  |  |  |
| Receiving oral sex (7) |  |  |  |  |  |  |
| Sexual intercourse (8) |  |  |  |  |  |  |

Q5.7 How has the COVID19 pandemic affected your sexuality?

________________________________________________________________

Q5.8 How would you describe your coming out experience?

- Extremely positive (1)
- Somewhat positive (2)
- Neither positive nor negative (3)
- Somewhat negative (4)
- Extremely negative (5)
- I have not come out (6)
- Not applicable (cisgender and heterosexual/straight) (8)

Q5.9 At approximately what age did you first come out or begin sharing your sexual orientation or gender identity? 

________________________________________________________________

Q5.10 In general, how masculine do you think you are?

- Not at all (1)
- Very little (2)
- Fairly (3)
- Very much (4)
- Extremely (5)

Q5.11 In general, how masculine do you act?

- Not at all (1)
- Very little (2)
- Fairly (3)
- Very much (4)
- Extremely (5)

Q5.12 How masculine do you think you appear to others?

- Not at all (1)
- Very little (2)
- Fairly (3)
- Very much (4)
- Extremely (5)

Q5.13 In general, how feminine do you think you are?

- Not at all (1)
- Very little (2)
- Fairly (3)
- Very much (4)
- Extremely (5)

Q5.14 In general, how feminine do you act?

- Not at all (1)
- Very little (2)
- Fairly (3)
- Very much (4)
- Extremely (5)

Q5.15 How feminine do you think you appear to others?

- Not at all (1)
- Very little (2)
- Fairly (3)
- Very much (4)
- Extremely (5)

End of Block: Sex & Gender

Start of Block: Physical and Behavioral Health

Q6.1 How tall are you (choose **either** feet & inches **or** centimeters)?

|  | Feet & Inches | Centimeters |
| --- | --- | --- |
|  |  |  |
| Your height: (1) | ▼ 0'0"  (1 ... 8'11" (108) | ▼ 0 (1 ... 271.78 (108) |

Q6.2 How much do you weigh (choose **either** lbs **or** kg)?

|  | lbs | kg |
| --- | --- | --- |
|  |  |  |
| Your weight: (1) | ▼ 0 (1 ... 404 (405) | ▼ 0 (1 ... 183.22 (405) |

Q6.3 Rate the level of satisfaction and fulfillment in your life.

- 0 -None (1)
- 1 (2)
- 2 (3)
- 3 (4)
- 4 (5)
- 5 -Extremely high (6)

Q6.4 How would you describe your physical health?

- Excellent (1)
- Very good (2)
- Good (3)
- Fair (4)
- Poor (5)

Q6.5 How would you describe your mental health?

- Excellent (1)
- Very good (2)
- Good (3)
- Fair (4)
- Poor (5)

Q6.6 What is your HIV/AIDS status?

- Positive (1)
- Positive, undetectable (2)
- Negative (3)
- Negative, on Pre-Exposure Prophylaxis (4)
- Unknown (5)

Display This Question:

If What is your HIV/AIDS status? = Positive

Or What is your HIV/AIDS status? = Positive, undetectable

Q6.7 Do you take any antiretroviral medication?

- Yes (1)
- No (2)

Q6.8 How often in the last year have you visited the emergency room or an urgent care centre?

- Daily or almost daily (1)
- Weekly (2)
- Monthly (3)
- Less than monthly (4)
- Never (5)

Q6.9 How would you rate the quality of health care services you have received this year?

- Excellent (1)
- Very good (2)
- Good (3)
- Fair (4)
- Poor (5)

Q6.10 Do you have a regular medical doctor (e.g. GP or Family doctor)?

- Yes (1)
- No (2)

Skip To: Q6.12 If Do you have a regular medical doctor (e.g. GP or Family doctor)? = Yes

Q6.11 Why do you not have a regular doctor?

- No medical doctors available in the area (1)
- Medical doctors in the area are not taking new patients (2)
- Have not tried to contact one (3)
- Had a medical doctor who left or retired (4)
- Other – Specify: (5) ________________________________________________

Display This Question:

If Do you have a regular medical doctor (e.g. GP or Family doctor)? = Yes

Q6.12 How often do you visit your regular medical doctor?

- Daily or almost daily (1)
- Weekly (2)
- Monthly (3)
- Less than monthly (4)
- Never (5)

Q6.13 Have you visited a mental health care professional in the past year such as a psychologist, psychiatrist, or social worker?

- Yes (1)
- No (2)

Skip To: End of Block If Have you visited a mental health care professional in the past year such as a psychologist, psych... = No

Q6.14 How often have you visited a mental health care professional in the past year?

- Daily or almost daily (1)
- Weekly (2)
- Monthly (3)
- Less than monthly (4)

End of Block: Physical and Behavioral Health

Start of Block: PHQ

Q7.1 Over the last 4 weeks, how much have you been bothered by any of the following problems?

|  | Not bothered (1) | Bothered a little (2) | Bothered a lot (3) |
| --- | --- | --- | --- |
| Stomach pain (1) |  |  |  |
| Back pain (2) |  |  |  |
| Pain in your arms, legs, or joints (knees, hips, etc.) (3) |  |  |  |
| Menstrual cramps or other problems with your periods (4) |  |  |  |
| Pain or problems during sexual intercourse (5) |  |  |  |
| Headaches (6) |  |  |  |
| Chest pain (7) |  |  |  |
| Dizziness (8) |  |  |  |
| Fainting spells (9) |  |  |  |
| Feeling your heart pound or race (10) |  |  |  |
| Shortness of breath (11) |  |  |  |
| Constipation, loose bowels, or diarrhea (12) |  |  |  |
| Nausea, gas, or indigestion (13) |  |  |  |

Q7.2 Over the last 2 weeks, how often have you been bothered by any of the following problems?

|  | Not at all (1) | Several days (2) | More than half the days (3) | Nearly everyday (4) |
| --- | --- | --- | --- | --- |
| Little interest or pleasure in doing things (1) |  |  |  |  |
| Feeling down, depressed, or hopeless (2) |  |  |  |  |
| Trouble falling or staying asleep, or sleeping too much (3) |  |  |  |  |
| Feeling tired or having little energy (4) |  |  |  |  |
| Poor appetite or overeating (5) |  |  |  |  |
| Feeling bad about yourself — or that you are a failure or have let yourself or your family and friends down (6) |  |  |  |  |
| Trouble concentrating on things, such as reading books or watching television (7) |  |  |  |  |
| Moving or speaking so slowly that other people could have noticed or the opposite — being so fidgety or restless that you have been moving around a lot more than usual (8) |  |  |  |  |

Q7.3 In the last 4 weeks, have you had an anxiety attack – suddenly feeling fear or panic?

- Yes (1)
- No (14)

Display This Question:

If In the last 4 weeks, have you had an anxiety attack – suddenly feeling fear or panic? = Yes

Q7.4 Regarding the anxiety attack you mentioned in the previous question: Has this ever happened before?

- Yes (1)
- No (2)

Display This Question:

If In the last 4 weeks, have you had an anxiety attack – suddenly feeling fear or panic? = Yes

Q7.5 Do some of these attacks come suddenly out of the blue, that is, in situations where you don’t expect to be nervous or uncomfortable?

- Yes (1)
- No (2)

Display This Question:

If In the last 4 weeks, have you had an anxiety attack – suddenly feeling fear or panic? = Yes

Q7.6 Do these attacks bother you a lot or are you worried about having another attack?

- Yes (1)
- No (2)

Display This Question:

If In the last 4 weeks, have you had an anxiety attack – suddenly feeling fear or panic? = Yes

Q7.7 Think about your last bad anxiety attack.

|  | No (1) | Yes (2) |
| --- | --- | --- |
| Were you short of breath? (1) |  |  |
| Did your heart race, pound, or skip? (2) |  |  |
| Did you have chest pain or pressure? (3) |  |  |
| Did you sweat? (4) |  |  |
| Did you feel as if you were choking? (5) |  |  |
| Did you have hot flashes or chills? (6) |  |  |
| Did you have nausea or an upset stomach, or the feeling that you were going to have diarrhea? (7) |  |  |
| Did you feel dizzy, unsteady, or faint? (8) |  |  |
| Did you have tingling or numbness in parts of your body? (9) |  |  |
| Did you tremble or shake? (10) |  |  |
| Were you afraid you were dying? (11) |  |  |

Q7.8 Over the last 4 weeks, how often have you been bothered by any of the following problems?

|  | Not at all (1) | Several days (2) | More than half the days (3) |
| --- | --- | --- | --- |
| Feeling nervous, anxious, on edge, or worrying a lot about different things (1) |  |  |  |
| Feeling restless so that it is hard to sit still (2) |  |  |  |
| Getting tired very easily (3) |  |  |  |
| Muscle tension, aches, or soreness (4) |  |  |  |
| Trouble falling asleep or staying asleep (5) |  |  |  |
| Trouble concentrating on things, such as reading a book or watching TV (6) |  |  |  |
| Becoming easily annoyed or irritable (7) |  |  |  |

Q7.9 The following questions ask you to think about your eating patterns.

|  | No (1) | Yes (2) |
| --- | --- | --- |
| Do you often feel that you can’t control what or how much you eat? (1) |  |  |
| Do you often eat, within any 2-hour period, what most people would regard as an unusually large amount of food? (2) |  |  |
| On average have either of the above happened as often as twice a week for the last 3 months? (3) |  |  |

Q7.10 In the last 3 months have you often done any of the following in order to avoid gaining weight?

|  | No (1) | Yes (2) |
| --- | --- | --- |
| Made yourself vomit? (1) |  |  |
| Took more than twice the recommended dose of laxatives? (2) |  |  |
| Fasted – not eaten anything at all for at least 24 hours? (3) |  |  |
| Exercised for more than an hour specifically to avoid gaining weight after binge eating? (4) |  |  |

Display This Question:

If In the last 3 months have you often done any of the following in order to avoid gaining weight? = Yes

Q7.11 If you checked “YES” to any of these ways of avoiding gaining weight, were any as often, on average, as twice a week?

- Yes (1)
- No (2)

Q7.12 If you checked off any problems on this questionnaire, how difficult have these problems made it for you to do your work, take care of things at home, or get along with other people?

- Not difficult at all (1)
- Somewhat difficult (2)
- Very difficult (3)
- Extremely difficult (4)

Display This Question:

If How would you describe your mental health? = Fair

Or How would you describe your mental health? = Poor

Or Over the last 2 weeks, how often have you been bothered by any of the following problems? = Several days

Or Over the last 2 weeks, how often have you been bothered by any of the following problems? = More than half the days

Or Over the last 2 weeks, how often have you been bothered by any of the following problems? = Nearly everyday

Or In the last 4 weeks, have you had an anxiety attack – suddenly feeling fear or panic? = Yes

Or Over the last 4 weeks, how often have you been bothered by any of the following problems? = Feeling nervous, anxious, on edge, or worrying a lot about different things [ Several days ]

Or Over the last 4 weeks, how often have you been bothered by any of the following problems? = Feeling nervous, anxious, on edge, or worrying a lot about different things [ More than half the days ]

Q7.13 One or more of your responses to the previous questions raises some concerns from our team about your well-being and your psychological health. Given the current situation, it is normal to feel stress, anxiety or psychological distress, and it is all the more normal to seek help if you feel the need. You are welcome to contact a member of our team at queer.covid.to@gmail.com with any questions or concerns.   We are concerned about your psychological well-being, which is why we have put together a list of resources that you can contact if you feel the need:   If you are in distress or experiencing a mental health crisis, you can get support over the phone by calling: ·       Gerstein Crisis Centre: 416-929-5200 ·       Trans Lifeline (for trans and gender non-conforming folks): 1-877-330-6366 ·       The Lesbian, Gay, Bi & Trans Youthline offers free peer support for youth aged 26 and under: 1-800-268-9688 ·       Toronto Distress Centre (24/7): 416-408-4357 or text 741741 (2am-2pm daily) ·       Toronto Rape Crisis Centre (24/7): 416-597-8808. ·       Assaulted Women's Helpline (24/7): 1-866-863-0511. ·       or visit your nearest Emergency Department   ·       For additional crisis support services in Toronto: https://toronto.cmha.ca/mental-health/find-help/ ·       For 24/7 distress lines across Ontario:  http://www.dcontario.org/centres.html   **Counselling Referrals:** ·       Crisis, Drop-in and Peer Supports in Toronto: https://www.the519.org/programs/referrals/crisis-drop-in-peer-supports ·       Counselling & Support Referrals: Youth and Young Adults: https://www.the519.org/programs/referrals/counselling-referrals-youth ·       Counselling Referrals for Adults 30+: https://www.the519.org/programs/referrals/counselling-referrals-30   **Additional Resources:** ·       COVID-19 & Emotional Self-Care Resources: https://www.the519.org/covid19-emotional-selfcare ·       The Lesbian, Gay, Bi & Trans Youthline: https://www.youthline.ca

End of Block: PHQ

Start of Block: The Ways of Coping Checklist

Q8.1 Consider a stressful situation that you have experienced in the past month which: was HIGHLY stressful (i.e. not something with a low degree of stress); you had NO CONTROL over (i.e. you could not prevent it from happening); and you COULD NOT PREDICT would happen (i.e. was unpredictable).


For each strategy below, indicate whether or not you used them to deal with this particular situation. Make sure to answer ALL questions. Do not leave any blank.

|  | Not used (1) | Used somewhat (2) | Used quite a bit (3) | Used a great deal (4) |
| --- | --- | --- | --- | --- |
| I made a plan of action and followed it. (1) |  |  |  |  |
| I wished I was a stronger person - more optimistic and forceful. (2) |  |  |  |  |
| I talked to someone about how I was feeling. (3) |  |  |  |  |
| I criticized or lectured myself. (4) |  |  |  |  |
| I changed or grew as a person in a good way. (5) |  |  |  |  |
| I just took things one step at a time. (6) |  |  |  |  |
| I wished that I could avoid the problem. (7) |  |  |  |  |
| I felt bad that I couldn't avoid the problem. (8) |  |  |  |  |
| I concentrated on something good that could come out of the whole thing. (9) |  |  |  |  |
| I talked to someone to find our more about the situation. (10) |  |  |  |  |
| I hoped a miracle would happen. (11) |  |  |  |  |
| I went on as if nothing happened. (12) |  |  |  |  |
| I changed something so things would turn out all right. (13) |  |  |  |  |
| I blamed myself. (14) |  |  |  |  |
| I kept my feelings to myself. (15) |  |  |  |  |
| I bargained or compromised to get something positive from the situation. (16) |  |  |  |  |
| I daydreamed or imagined a better time or place than the one I was in. (17) |  |  |  |  |
| I tried not to act too hastily or follow my own hunch. (18) |  |  |  |  |
| I refused to believe it had happened. (19) |  |  |  |  |
| I realized I brought the problem on myself. (20) |  |  |  |  |
| I tried not to isolate myself. (21) |  |  |  |  |
| I thought about fantastic or unreal things (like perfect revenge or finding a million dollars) that made me feel better. (22) |  |  |  |  |
| I accepted sympathy and understanding from someone. (23) |  |  |  |  |
| I came up with a couple of different solutions to the problem. (24) |  |  |  |  |
| I tried to forget the whole thing. (25) |  |  |  |  |
| I wished I could change the way I felt. (26) |  |  |  |  |
| I knew what had to be done so I doubled my efforts and tried harder to make things work. (27) |  |  |  |  |
| I changed something about myself so I could deal with the situation better. (28) |  |  |  |  |
| I tried not to act too hastily or follow my first hunch. (29) |  |  |  |  |
| I accepted the next best thing to do what I wanted. (30) |  |  |  |  |
| I came out of the experience better than when I went in. (31) |  |  |  |  |
| I accepted my strong feelings, but didn't let them interfere with other things too much. (32) |  |  |  |  |
| I stood my ground and fought for what I wanted. (33) |  |  |  |  |
| I got professional help and did what they recommended. (34) |  |  |  |  |
| I talked to someone who could do something about the problem. (35) |  |  |  |  |
| I asked someone I respected for advice and followed it. (36) |  |  |  |  |
| I had fantasies or wishes about how things might turn out. (37) |  |  |  |  |
| I wished the situation would go away or somehow be finished. (38) |  |  |  |  |
| I slept more than usual. (39) |  |  |  |  |
| I got mad at the people or things that caused the problem. (40) |  |  |  |  |
| I tried to make myself feel better by eating, drinking, smoking, or taking medication. (41) |  |  |  |  |
| I avoided being with people in general. (42) |  |  |  |  |
| I kept others from knowing how bad things were. (43) |  |  |  |  |

End of Block: The Ways of Coping Checklist

Start of Block: Global Physical Activity Questionnaire

Q9.1

These questions ask about the time you spend doing different types of physical activity in a typical week during the COVID-19 pandemic, lockdown, and quarantine. Please answer these questions even if you do not consider yourself to be a physically active person. Think first about the time you spend doing work. Think of work as the things that you have to do such as paid or unpaid work, study/training, household chores, harvesting food/crops, fishing or hunting for food, or seeking employment. In answering the following questions 'vigorous-intensity activities' are activities that require hard physical effort and cause large increases in breathing or heart rate, 'moderate-intensity activities' are activities that require moderate physical effort and cause small increases in breathing or heart rate.


Does your work involve vigorous-intensity activity that causes large increases in breathing or heart rate like carrying or lifting heavy loads, digging or construction work for at least 10 minutes continuously?

- Yes (1)
- No (2)

Skip To: Q9.4 If = No

Q9.2
In a typical week during the COVID-19 pandemic, on how many days do you do vigorous-intensity activities as part of your work?

________________________________________________________________

Q9.3
How much time do you spend doing vigorous-intensity activities at work on a typical day during the COVID-19 pandemic?

Hours (6)

Minutes (7)

▼ 0 (1) ... 23 ~ 59 (1464)

Q9.4
Does your work involve moderate-intensity activity, that causes small increases in breathing or heart rate such as brisk walking or carrying light loads for at least 10 minutes continuously?

- Yes (1)
- No (2)

Skip To: Q9.7 If Does your work involve moderate-intensity activity, that causes small increases in breathing or h... = No

| 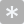 |
| --- |

Q9.5
In a typical week during the COVID-19 pandemic, on how many days do you do moderate-intensity activities as part of your work?

________________________________________________________________

Q9.6
How much time do you spend doing moderate-intensity activities at work on a typical day during the COVID-19 pandemic?

Hours (6)

Minutes (7)

▼ 0 (1) ... 23 ~ 59 (1464)

Q9.7

The next questions exclude the physical activities at work that you have already mentioned. They ask you about the usual way you travel to and from places. For example, to work, for shopping, to market, and to place of worship.


Do you walk or use a bicycle (pedal cycle) for at least 10 minutes continuously to get to and from places?

- Yes (1)
- No (2)

Skip To: Q9.10 If The next questions exclude the physical activities at work that you have already mentioned. They... = No

| 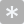 |
| --- |

Q9.8
In a typical week during the COVID-19 pandemic, on how many days do you walk or bicycle for at least 10 minutes continuously to get to and from places?

________________________________________________________________

Q9.9
How much time do you spend walking or bicycling for travel on a typical day during the COVID-19 pandemic?

Hours (6)

Minutes (7)

▼ 0 (1) ... 23 ~ 59 (1464)

Q9.10

The next questions exclude the work and transport activities that you have already mentioned. They ask you about sports, fitness, and recreational activities (leisure).


Do you do any vigorous-intensity sports, fitness or recreational (leisure) activities that cause large increases in breathing or heart rate like running or football for at least 10 minutes continuously?

- Yes (1)
- No (2)

Skip To: Q9.13 If The next questions exclude the work and transport activities that you have already mentioned. The... = No

Q9.11
In a typical week during the COVID-19 pandemic, on how many days do you do vigorous-intensity sports, fitness or recreational (leisure) activities?

________________________________________________________________

Q9.12
How much time do you spend doing vigorous-intensity sports, fitness or recreational activities on a typical day during the COVID-19 pandemic?

Hours (6)

Minutes (7)

▼ 0 (1) ... 23 ~ 59 (1464)

Q9.13
Do you do any moderate-intensity sports, fitness or recreational (leisure) activities that cause a small increase in breathing or heart rate such as brisk walking, cycling, swimming, volleyball, for at least 10 minutes continuously?

- Yes (1)
- No (2)

Skip To: Q9.16 If Do you do any moderate-intensity sports, fitness or recreational (leisure) activities that cause... = No

| 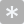 |
| --- |

Q9.14
In a typical week during the COVID-19 pandemic, on how many days do you do moderate-intensity sports, fitness or recreational (leisure) activities?

________________________________________________________________

Q9.15
How much time do you spend doing moderate-intensity sports, fitness or recreational (leisure) activities on a typical day during the COVID-19 pandemic?

Hours (6)

Minutes (7)

▼ 0 (1) ... 23 ~ 59 (1464)

Q9.16

The following question is about sitting or reclining at work, at home, getting to and from places, or with friends including time spent sitting at a desk, sitting with friends, traveling in a car, bus, train, reading, playing cards or watching television, but do not include time spent sleeping.


How much time do you usually spend sitting or reclining on a typical day during the COVID-19 pandemic?

Hours (6)

Minutes (7)

▼ 0 (1) ... 23 ~ 59 (1464)

End of Block: Global Physical Activity Questionnaire

Start of Block: The Pittsburgh Sleep Quality Index

Q10.1 During the COVID-19 pandemic, what time have you usually gone to bed at night? Please use a 24h format, such that 9am = 09:00 and 9pm = 21:00.

________________________________________________________________

| 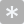 |
| --- |

Q10.2 During the COVID-19 pandemic, how long (in minutes) has it usually taken you to fall asleep each night? In other words, this means the time between when you turn the lights out and when you fall asleep. For example, if you turn the lights out at 11:00 pm and you fall asleep around 11:15, your answer is 15.

________________________________________________________________

Q10.3 During the COVID-19 pandemic, what time have you usually gotten up in the morning? Please use a 24h format such that 9am = 09:00 and 9pm = 21:00.

________________________________________________________________

Q10.4 During the COVID-19 pandemic, how many hours of actual sleep do you generally get at night? This is the number of hours asleep between when you fall asleep and wake up, and may be shorter than the number of hours you spend in bed.

________________________________________________________________

Q10.5 During the COVID-19 pandemic, how often have you had trouble sleeping because you:

|  | Not during the past month (1) | Less than once a week (2) | Once or twice a week (3) | Three or more times a week (4) |
| --- | --- | --- | --- | --- |
| Cannot get to sleep within 30 minutes (1) |  |  |  |  |
| Wake up in the middle of the night or early morning (2) |  |  |  |  |
| Have to get up to use the bathroom (3) |  |  |  |  |
| Cannot breathe comfortably (4) |  |  |  |  |
| Cough or snore loudly (5) |  |  |  |  |
| Feel too cold (6) |  |  |  |  |
| Feel too hot (7) |  |  |  |  |
| Have bad dreams (8) |  |  |  |  |
| Have pain (9) |  |  |  |  |
| Other (Specify): (10) |  |  |  |  |

Q10.6 During the COVID-19 pandemic, how would you rate your sleep quality overall?

- Very good (1)
- Fairly good (2)
- Fairly bad (3)
- Very bad (4)

Q10.7 During the COVID-19 pandemic, how often have you taken medicine (prescribed or "over the counter") to help you sleep?

- Not during the past month (1)
- Less than once a week (2)
- Once or twice a week (3)
- Three or more times a week (4)

Q10.8 During the COVID-19 pandemic, how often have you had trouble staying awake while driving, eating meals, or engaging in social activity?

- Not during the past month (1)
- Less than once a week (2)
- Once or twice a week (3)
- Three or more times a week (4)

Q10.9 During the past month, how much of a problem has it been for you to keep up enough enthusiasm to get things done?

- No problem at all (1)
- Only a slight problem (2)
- Somewhat of a problem (3)
- A very big problem (4)

End of Block: The Pittsburgh Sleep Quality Index

Start of Block: Tobacco, Alcohol & Substance Use

Q11.1 Do you currently smoke cigarettes?

- Yes (1)
- No (2)

Skip To: Q11.4 If Do you currently smoke cigarettes? = No

Q11.2 Do you smoke everyday?

- Yes (how many do you smoke on average daily?) (1) ________________________________________________
- No (2)

Skip To: Q11.5 If Do you smoke everyday? = Yes (how many do you smoke on average daily?)

Q11.3 Do you smoke only occasionally?

- Yes (on average, how many cigarettes do you smoke per week?) (1) ________________________________________________
- No (2)

Display This Question:

If Do you currently smoke cigarettes? = No

Q11.4 Did you ever smoke cigarettes regularly in the past?

- Yes (1)
- No (2)

Q11.5 Do you currently use cannabis?

- Yes (1)
- No (2)

Skip To: Q11.7 If Do you currently use cannabis?  = No

Q11.6 How often do you use cannabis?

- Daily (how many times daily?) (1) ________________________________________________
- Occasionally (2)
- Never (3)

Q11.7 How often do you have a drink containing alcohol (including beer or wine)?

- Never (1)
- Monthly or less (2)
- 2-4 times a month (3)
- 2-3 times a week (4)
- 4 or more times a week (5)

Skip To: Q11.12 If How often do you have a drink containing alcohol (including beer or wine)? = Never

Q11.8 How many drinks do you have on a typical day when you are drinking?

- 1 or 2 (1)
- 3 or 4 (2)
- 5 or 6 (3)
- 7 to 9 (4)
- 10 or more (5)

Q11.9 The following questions ask you to think about your alcohol use.

|  | Never (1) | Less than monthly (2) | Monthly (3) | Weekly (4) | Daily or almost daily (5) |
| --- | --- | --- | --- | --- | --- |
| How often do you have six or more drinks on one occasion? (1) |  |  |  |  |  |
| How often during the last year have you found that you were not able to stop drinking once you started? (2) |  |  |  |  |  |
| How often during the last year have you failed to do what was normally expected of you because of drinking? (3) |  |  |  |  |  |
| How often during the last year have you needed a drink first in the morning to get yourself going after a heavy drinking session? (4) |  |  |  |  |  |
| How often during the last year have you felt guilt or remorse after drinking? (5) |  |  |  |  |  |
| How often during the last year have you been unable to remember what happened the night before because of your drinking? (6) |  |  |  |  |  |

Q11.10 Have you or someone else been injured because of your drinking?

- No (1)
- Yes, but not in the last year (2)
- Yes, during the last year (3)

Q11.11 Has a relative, friend, doctor, or health care worker been concerned about your drinking or suggested you cut down?

- No (1)
- Yes, but not in the last year (2)
- Yes, during the last year (3)

Q11.12 How often do you use drugs other than alcohol?

- Never (2)
- Monthly or less (6)
- 2-4 times a month (3)
- 2-3 times a week (4)
- 4 or more times a week (5)

Skip To: End of Block If How often do you use drugs other than alcohol? = Never

Q11.13 How often do you use more than one type of drug on the same occasion?

- Never (1)
- Monthly or less (2)
- 2-4 times a month (3)
- 2-3 times a week (4)
- 4 or more times a week (5)

Q11.14 How many times do you take drugs on a typical day when you use drugs?

- 1 or 2 (1)
- 3 or 4 (2)
- 5 or 6 (3)
- 7 to 9 (4)
- 10 or more (5)

End of Block: Tobacco, Alcohol & Substance Use

Start of Block: Perceived Stress Scale

Q12.1 In the last month, how often have you:

|  | Never (1) | Almost never (2) | Sometimes (3) | Fairly often (4) | Very Often (5) |
| --- | --- | --- | --- | --- | --- |
| Been upset because of something that happened unexpectedly? (1) |  |  |  |  |  |
| Felt that you were unable to control the important things in your life? (2) |  |  |  |  |  |
| Felt nervous and "stressed"? (3) |  |  |  |  |  |
| Dealt successfully with irritating life hassles? (4) |  |  |  |  |  |
| Felt that you were effectively coping with important changes were occurring in your life? (5) |  |  |  |  |  |
| Felt confident about your ability to handle your personal problems? (6) |  |  |  |  |  |
| Felt that things were going your way? (7) |  |  |  |  |  |
| Found that you could not cope with all the things that you had to do? (8) |  |  |  |  |  |
| Been able to control irritations in your life? (9) |  |  |  |  |  |
| Felt that you were on top of things? (10) |  |  |  |  |  |
| Been angered because of things that happened that were outside of your control? (11) |  |  |  |  |  |
| Found yourself thinking about things that you have to accomplish? (12) |  |  |  |  |  |
| Been able to control the way you spend your time? (13) |  |  |  |  |  |
| Felt difficulties were piling up so high that you could not overcome them? (14) |  |  |  |  |  |

Q12.2 Thinking about stress in your day-to-day life, what would you say is the most important thing contributing to feelings of stress you may have?

- Time pressures / not enough time (1)
- My own physical health problem or condition (2)
- My own emotional or mental health problem or condition (3)
- Financial situation (e.g., not enough money, debt) (4)
- My own work situation (e.g., hours of work, working conditions) (5)
- School (6)
- Employment status (e.g., unemployment) (7)
- Caring for my children (8)
- Caring for other family members or friends (9)
- Other personal or family responsibilities (10)
- Personal relationships (11)
- Discrimination (12)
- My personal safety and/or my family's personal safety (13)
- Health of family members (14)
- Other – Specify: (15) ________________________________________________
- Nothing (16)

| 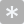 |
| --- |

Q12.3 On a scale from 0 (not stressed) to 10 (overwhelmed) how stressed have you felt, on average, over the past three months?

________________________________________________________________

End of Block: Perceived Stress Scale

Start of Block: GSS Social Identity

| 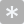 |
| --- |

Q13.1 How many relatives (including your parents, siblings, aunts, uncles, cousins, and in-laws) do you have who you feel close to, that is, who you feel at ease with, can talk to about what is on your mind, or call on for help?

________________________________________________________________

| 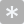 |
| --- |

Q13.2 How many friends do you have (people who are not your relatives), who you feel at ease with, can talk to about what is on your mind, or call on for help?

________________________________________________________________

Q13.3 Thinking of all the relatives (including your parents, siblings, aunts, uncles, cousins, and in-laws) you had contact with in the past year, how often did you see any of your **relatives** in person? Exclude people you live with.

- Every day (1)
- A few times a week (2)
- Once a week (7)
- 2 or 3 times a month (3)
- Once a month (5)
- Not in the past month (6)

Q13.4 Thinking of all the relatives (including your parents, siblings, aunts, uncles, cousins, and in-laws) you had contact with in the past year, how often did you communicate with any of your relatives by telephone, text message, or online social networks? Include all forms of Internet communication (e.g., Facebook, Twitter, instant message, Skype and FaceTime.) Exclude people you live with.

- Every day (1)
- A few times a week (2)
- Once a week (3)
- 2 or 3 times a week (4)
- Once a month (5)
- Not in the past month (6)

Q13.5 Overall, how satisfied are you with how often you communicate with your relatives?

- Very satisfied (1)
- Satisfied (2)
- Neither satisfied nor dissatisfied (3)
- Dissatisfied (4)
- Very dissatisfied (5)

Display This Question:

If Overall, how satisfied are you with how often you communicate with your relatives? = Dissatisfied

Or Overall, how satisfied are you with how often you communicate with your relatives? = Very dissatisfied

Q13.6 Are you dissatisfied because you communicate with your family members (including your parents, siblings, aunts, uncles, cousins, and in-laws) too often or not often enough?

- Too often (1)
- Not enough (2)

Q13.7 Thinking of all the friends you had contact with in the past month, how often did you see any of your friends in person? Exclude people you live with.

- Every day (1)
- A few times a week (2)
- Once a week (3)
- 2 or 3 times a month (4)
- Once a month (5)
- Not in the past month (6)

Q13.8 Thinking of all the friends you had contact with in the past month, how often did you communicate with any of your friends by telephone, text message, or online social networks? Include all forms of Internet communication (e.g., Facebook, Twitter, instant message, Skype and FaceTime). Exclude people you live with.

- Every day (1)
- A few times a week (2)
- Once a week (3)
- 2 or 3 times a month (4)
- Once a month (5)
- Not in the past month (6)

Q13.9 Overall, how satisfied are you with how often you communicate with your friends?

- Very satisfied (1)
- Satisfied (2)
- Neither satisfied nor dissatisfied (3)
- Dissatisfied (4)
- Very dissatisfied (5)

Display This Question:

If Overall, how satisfied are you with how often you communicate with your friends? = Dissatisfied

Or Overall, how satisfied are you with how often you communicate with your friends? = Very dissatisfied

Q13.10 Are you dissatisfied because you communicate with your friends too often or not often enough?

- Too often (1)
- Not enough (2)

Q13.11 In the past 12 months, were you a member or participant in the following groups, organizations or associations? These could be formally organized groups or just groups of people who get together regularly to do an activity or talk about things. Include groups you are active in through the Internet.

|  | Yes (1) | No (2) |
| --- | --- | --- |
| Sports or recreational organization (e.g. hockey league, health club or golf club) (1) |  |  |
| Cultural, educational or hobby organization (e.g. theatre group, book club or bridge club) (2) |  |  |
| Union or professional association (3) |  |  |
| Political party or group (4) |  |  |
| Religious-affiliated group (Exclude regular attendance at your place of worship.) (5) |  |  |
| School group, neighbourhood, civic or community association (e.g. parent-teacher association (PTA), alumni association, block parents or neighbourhood watch) (6) |  |  |
| Humanitarian or charitable organization or service club (e.g. Meals on Wheels, United Way, Unicef, Heart and Stroke Foundation, Distress Centre, Rotary Club, Red Cross) (7) |  |  |
| Seniors' group (e.g. seniors' club, recreational association or resource centre) (8) |  |  |
| Youth organization (e.g. Me to We, Scouts, Guides, Big Brothers or Big Sisters, YMCA or YWCA) (9) |  |  |
| Immigrant or ethnic association or club (10) |  |  |
| Environmental group (e.g. in the areas of conservation, ecology, the environment or animal rights) (11) |  |  |
| Other type of group, organization or association (Specify other type of group, organization or association) (12) |  |  |

| 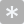 |
| --- |

Q13.12 Of all the types of groups, organizations or associations in the previous question, in how many were you a member or participant in the past 12 months?

________________________________________________________________

| 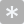 |
| --- |

Q13.13 How many of these groups were you active in through the Internet? Include any involvement through the Internet in the past 12 months, whether you conducted part or all of your activities through the Internet. Your answer should be based on what "being active through the Internet" means to you.

________________________________________________________________

Q13.14 How often did you participate in group activities and meetings on the Internet? Exclude group activities and meetings in person.

- At least once a week (1)
- A few times a month (2)
- Once a month (3)
- Once or twice a year (4)
- Not in the past year (5)

Q13.15 How often did you participate in group activities and meetings in person? Exclude group activities and meetings on the Internet.

- At least once a week (1)
- A few times a month (2)
- Once a month (3)
- Once or twice a year (4)
- Not in the past year (5)

Q13.16 Over the past five years, would you say that your involvement in organizations has increased, decreased, or stayed the same?

- Increased (1)
- Decreased (2)
- Stayed the same (3)

End of Block: GSS Social Identity

Start of Block: Perceived Vulnerability to Disease

Q14.1 The following questions ask you to think how vulnerable you feel when it comes to illness and disease. 

|  | Strongly agree (1) | Agree (2) | Somewhat agree (3) | Neither agree nor disagree (4) | Somewhat disagree (5) | Disagree (6) | Strongly disagree (7) |
| --- | --- | --- | --- | --- | --- | --- | --- |
| In general, I am very susceptible to colds, flu and other infectious diseases. (1) |  |  |  |  |  |  |  |
| If an illness is ‘going around’, I will get it. (2) |  |  |  |  |  |  |  |
| My immune system protects me from most illnesses that other people get. (3) |  |  |  |  |  |  |  |
| I am more likely than the people around me to catch an infectious disease. (4) |  |  |  |  |  |  |  |
| My past experiences make me believe I am not likely to get sick even when my friends are sick. (5) |  |  |  |  |  |  |  |
| I have a history of susceptibility to infectious disease. (6) |  |  |  |  |  |  |  |
| I prefer to wash my hands pretty soon after shaking someone’s hand. (7) |  |  |  |  |  |  |  |
| I avoid touching other people's cell phones because of the risk that I may catch something from the previous user. (8) |  |  |  |  |  |  |  |
| I do not like to write with a pencil someone else has obviously chewed on. (9) |  |  |  |  |  |  |  |
| I dislike wearing used clothes because you do not know what the last person who wore it was like. (10) |  |  |  |  |  |  |  |
| I am comfortable sharing a water bottle with a friend. (11) |  |  |  |  |  |  |  |
| It really bothers me when people sneeze without covering their mouths (12) |  |  |  |  |  |  |  |
| It does not make me anxious to be around sick people. (13) |  |  |  |  |  |  |  |
| My hands do not feel dirty after touching money. (14) |  |  |  |  |  |  |  |
| I am unlikely to catch a cold, flu or other illness, even if it is “going around (15) |  |  |  |  |  |  |  |
| 16 (16) |  |  |  |  |  |  |  |

End of Block: Perceived Vulnerability to Disease

Start of Block: COVID-19 Health and Experience Scale

Q15.1 During the COVID-19 pandemic, have you been exposed to someone who has or is likely to have COVID-19? (check all that apply)

- Yes, someone with a positive test (1)
- Yes, someone with a medical diagnosis, but no test (2)
- Yes, someone with possible symptoms but no test or medical diagnosis (3)
- No, not to my knowledge (4)

Q15.2 During the COVID-19 pandemic, have you had a COVID-19 infection?

- Yes, with a positive test (1)
- Yes, with a medical diagnosis, but no test (2)
- Yes, with possible symptoms but no test or medical diagnosis (3)
- No, not to my knowledge (4)

Q15.3 During the COVID-19 pandemic, have you had any of the following symptoms? (check all that apply)

- Fever (1)
- Cough (2)
- Shortness of breath (3)
- Sore throat (4)
- Fatigue (5)
- Loss of taste or smell (6)
- Eye infection (7)
- Other (8) ________________________________________________
- None (9)

Q15.4 During the COVID-19 pandemic, have any of the following happened to you because of COVID-19? (check all that apply)

- Fallen ill physically (1)
- Hospitalized (2)
- Put into self-quarantine with symptoms (3)
- Put into self-quarantine without symptoms (e.g., due to possible exposure) (4)
- Lost or been laid off from job (5)
- Reduced ability to earn money (6)
- None of the above (8)

Q15.5 During the COVID-19 pandemic has anyone in your family been diagnosed with COVID-19? (check all that apply)

- Yes, a member of my household (1)
- Yes, a non-household member of my family (2)
- No (3)

Q15.6 During the COVID-19 pandemic, have any of the following happened to your family/bubble members because of COVID-19? (check all that apply)

- Fallen ill physically (1)
- Hospitalized (2)
- Put into self-quarantine with symptoms (3)
- Put into self-quarantine without symptoms (e.g., due to possible exposure) (4)
- Lost or been laid off from job (5)
- Reduced ability to earn money (6)
- Passed away (7)
- None of the above (8)

Q15.7 During the COVID-19 pandemic, how worried have you been about:

|  | Not at all (1) | Slightly (2) | Moderately (3) | Very (4) | Extremely (5) |
| --- | --- | --- | --- | --- | --- |
| Being infected (1) |  |  |  |  |  |
| Friends or family being infected (2) |  |  |  |  |  |
| Your physical health being influenced by Coronavirus/COVID-19 (3) |  |  |  |  |  |
| Your mental/emotional health being influenced by COVID-19? (4) |  |  |  |  |  |
| Other (please specifiy): (5) |  |  |  |  |  |

Q15.8 How much are you reading or talking about COVID-19?

- Never (1)
- Rarely (2)
- Occasionally (3)
- Often (4)
- Most of the Time (5)

| 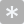 |
| --- |

Q15.9 During the COVID-19 pandemic, how many people from outside of your household have you had an in-person interaction with?

________________________________________________________________

Q15.10 During the COVID-19 pandemic, how often have you gone outside of the home (e.g., going to stores, parks, etc.)?

- Not at all (1)
- 1-2 days per week (2)
- A few days per week (3)
- Several days per week (4)
- Every day (5)

Q15.11 During the COVID-19 pandemic, how stressful have the restrictions on leaving home been for you?

- Extremely (1)
- Very (2)
- Moderately (3)
- Slightly (4)
- Not at all (5)

Q15.12 During the COVID-19 pandemic, how often have you had in-person contact with people outside of your household?

- A lot less than before COVID-19 (1)
- A little less than before COVID-19 (2)
- About the same as before COVID-19 (3)
- A little more than before COVID-19 (4)
- A lot more than before COVID-19 (5)

Q15.13 During the COVID-19 pandemic, how much difficulty have you had following the recommendations for avoiding close contact with people?

- None (1)
- A little (2)
- Moderate (3)
- A lot (4)

Q15.14 During the COVID-19 pandemic how has the quality of the relationships between you and members of your family changed? Have these relationships generally:

- Become a lot worse (1)
- Become a little worse (2)
- Stayed about the same (3)
- Become a little better (4)
- Become a lot better (5)

Q15.15 Do you currently feel lonely?

- Yes (1)
- No (2)

Q15.16 Compared to before COVID-19, how often do you feel lonely?

- A lot less than before COVID-19 (1)
- Somewhat less than before COVID-19 (2)
- The same as before COVID-19 (3)
- Somewhat more than before COVID-19 (4)
- A lot more than before COVID-19 (5)

Q15.17 Has the COVID-19 crisis in your area led to any positive changes in your life?

- None (1)
- Only a few. Please specify: (2) ________________________________________________
- Some. Please specify: (3) ________________________________________________
- Many. Please specify: (4) ________________________________________________

End of Block: COVID-19 Health and Experience Scale

Start of Block: Perceived Social Support Scale

Q16.1 Please indicate the extent to which you agree or disagree with the following statements:

|  | Strongly Disagree (1) | Generally Disagree (2) | Generally Agree (3) | Strongly Agree (4) |
| --- | --- | --- | --- | --- |
| My friends give me the moral support I need. (1) |  |  |  |  |
| Most other people are closer to their friends than I am. (2) |  |  |  |  |
| My friends enjoy hearing about what I think. (3) |  |  |  |  |
| Certain friends come to see me when they have problems or need advice. (4) |  |  |  |  |
| I rely on my friends for emotional support. (5) |  |  |  |  |
| If I feel that one or more of my friends are upset with me, I just keep it to myself. (6) |  |  |  |  |
| I feel that I’m not welcome in my circle of friends. (7) |  |  |  |  |
| I have a friend I could go to if I were feeling down, and wouldn’t feel embarrassed. (8) |  |  |  |  |
| My friends and I are very open about how we feel about things. (9) |  |  |  |  |
| My friends are sensitive to my personal needs. (10) |  |  |  |  |
| My friends come to me for emotional support. (11) |  |  |  |  |
| My friends are good at helping me solve problems. (12) |  |  |  |  |
| I have a close relationship with a number of friends (13) |  |  |  |  |
| My friends get good ideas about how to do things or make things from me. (14) |  |  |  |  |
| When I confide in friends, it makes me feel uncomfortable. (15) |  |  |  |  |
| My friends seek me out for companionship (16) |  |  |  |  |
| I think that my friends feel that I’m good at helping them solve problems. (17) |  |  |  |  |
| I don’t have a relationship with a friend that is as intimate as other peoples’ relationships with friends. (18) |  |  |  |  |
| I’ve recently gotten a good idea about how to do something from a friend. (19) |  |  |  |  |
| I wish my friends were much different. (20) |  |  |  |  |

End of Block: Perceived Social Support Scale

Start of Block: Social Isolation Survey Items

Q17.1 The next questions are about how you feel about different aspects of your life. For each one, select how often you feel that way. How often do you:

|  | Hardly Ever (1) | Some of the time (2) | Often (3) |
| --- | --- | --- | --- |
| Feel that you lack companionship? (1) |  |  |  |
| Feel left out? (2) |  |  |  |
| Feel isolated from others? (3) |  |  |  |

End of Block: Social Isolation Survey Items

Start of Block: The Everyday Discrimination Scale

Q18.1 Please respond to the following statements.

|  | Never (1) | Less than once a year (2) | A few times a year (3) | A few times a month (4) | At least once a week (5) | Almost everyday (6) |
| --- | --- | --- | --- | --- | --- | --- |
| You are treated with less courtesy than other people are. (1) |  |  |  |  |  |  |
| You are treated with less respect than other people are. (2) |  |  |  |  |  |  |
| You receive poorer service than other people at restaurants or stores. (3) |  |  |  |  |  |  |
| People act is if they think you are not smart. (4) |  |  |  |  |  |  |
| People act as if they are afraid of you. (5) |  |  |  |  |  |  |
| People act as if they think you are dishonest. (6) |  |  |  |  |  |  |
| People act as if they are better than you are. (7) |  |  |  |  |  |  |
| You are called names or insulted. (8) |  |  |  |  |  |  |
| You are threatened or harassed. (9) |  |  |  |  |  |  |

Q18.2 In the following questions, we are interested in the way other people have treated you and your beliefs about how other people have treated you. Please answer whether any of the following has ever happened to you:

|  | Yes (1) | No (2) | Don't know/prefer not to answer (3) |
| --- | --- | --- | --- |
| Have you ever not been hired for a job for unfair reasons? (1) |  |  |  |
| Have you ever been unfairly denied a promotion? (2) |  |  |  |
| Have you ever been unfairly stopped, searched, questioned, physically threatened, or abused by the police? (3) |  |  |  |
| Have you ever been unfairly discouraged by a teacher or advisor from continuing your education? (4) |  |  |  |
| Have you ever been unfairly prevented from moving into a neighbourhood because the landlord or realtor refused to sell or rent you a house or an apartment? (5) |  |  |  |
| Have you ever moved into a neighbourhood where neighbours made life difficult for you or your family? (6) |  |  |  |
| Have you ever been unfairly denied a bank loan? (7) |  |  |  |
| Have you ever received service from someone such as a plumber or car mechanic that was worse than what other people get? (8) |  |  |  |

Display This Question:

If In the following questions, we are interested in the way other people have treated you and your b... = Yes

Or Please respond to the following statements. = Less than once a year

Or Please respond to the following statements. = A few times a year

Or Please respond to the following statements. = A few times a month

Or Please respond to the following statements. = At least once a week

Or Please respond to the following statements. = Almost everyday

Q18.3 What do you think the reasons might be for you to have had these experiences? Was it ... (Mark all that apply).

- Your ancestry or national origins (1)
- Your gender (2)
- Your race (3)
- Your age (4)
- Your religion (5)
- Your height (6)
- Your weight (7)
- Some other aspect of your physical appearance (8)
- Your sexual orientation (9)
- Your education or income level (10)
- Other (11) ________________________________________________

Display This Question:

If In the following questions, we are interested in the way other people have treated you and your b... = Yes

Q18.4 Of the reasons you just mentioned, which one do you think is the main reason?

- Your ancestry or national origins (1)
- Your gender (2)
- Your race (3)
- Your age (4)
- Your religion (5)
- Your height (6)
- Your weight (7)
- Some other aspect of your physical appearance (8)
- Your sexual orientation (9)
- Your education or income level (10)
- Other (11) ________________________________________________

Display This Question:

If In the following questions, we are interested in the way other people have treated you and your b... = Yes

Q18.5 When was the last time this happened?

- Within the past week (1)
- Within the past month (2)
- Within the past year (3)
- More than a year ago (4)

Display This Question:

If In the following questions, we are interested in the way other people have treated you and your b... = Yes

| 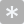 |
| --- |

Q18.6 How many times has this happened during your lifetime?

________________________________________________________________

End of Block: The Everyday Discrimination Scale

Start of Block: CCHS Household Food Security Survey

Q19.1 Which of the following statements best describes the food eaten in your household in the past 12 months, that is since April of last year?

- You and other household members always had enough of the kinds of foods you wanted to eat (1)
- You and other household members had enough to eat, but not always the kinds of food you wanted (2)
- Sometimes you and other household members did not have enough to eat (3)
- Often you and other household members didn't have enough to eat (4)
- Don't know (5)
- Prefer not to answer (6)

Skip To: End of Block If Which of the following statements best describes the food eaten in your household in the past 12... = Don't know

Skip To: End of Block If Which of the following statements best describes the food eaten in your household in the past 12... = Prefer not to answer

Q19.2 You and other household members worried that food would run out before you got money to buy more. Was that often true, sometimes true, or never true in the past 12 months?

- Often true (1)
- Sometimes true (2)
- Never true (3)
- Don't know (4)
- Prefer not to answer (5)

Q19.3 The food that you and other household members bought just didn't last, and there wasn't any money to get more. Was that often true, sometimes true, or never true in the past 12 months?

- Often true (1)
- Sometimes true (2)
- Never true (3)
- Don't know (4)
- Prefer not to answer (5)

Q19.4 You and other household members couldn't afford to eat balanced meals. In the past 12 months was that often true, sometimes true, or never true?

- Often true (1)
- Sometimes true (2)
- Never true (3)
- Don't know (4)
- Prefer not to answer (5)

Q19.5 Are there children under 18 in your household?

- Yes (1)
- No (2)

Skip To: Q19.9 If Are there children under 18 in your household? = No

Q19.6 You or other adults in your household relied on only a few kinds of low-cost food to feed the child(ren) because you were running out of money to buy food. Was that often true, sometimes true, or never true in the past 12 months?

- Often true (1)
- Sometimes true (2)
- Never true (3)
- Don't know (4)
- Prefer not to answer (5)

Q19.7 You or other adults in your household couldn't feed the child(ren) a balanced meal, because you couldn't afford it. Was that often true, sometimes true, or never true in the past 12 months?

- Often true (1)
- Sometimes true (2)
- Never true (3)
- Don't know (4)
- Prefer not to answer (5)

Display This Question:

If Which of the following statements best describes the food eaten in your household in the past 12... = Sometimes you and other household members did not have enough to eat

Or Which of the following statements best describes the food eaten in your household in the past 12... = Often you and other household members didn't have enough to eat

Or You and other household members worried that food would run out before you got money to buy more.... = Often true

Or You and other household members worried that food would run out before you got money to buy more.... = Sometimes true

Or The food that you and other household members bought just didn't last, and there wasn't any money... = Often true

Or The food that you and other household members bought just didn't last, and there wasn't any money... = Sometimes true

Or You and other household members couldn't afford to eat balanced meals. In the past 12 months was... = Often true

Or You and other household members couldn't afford to eat balanced meals. In the past 12 months was... = Sometimes true

Or You or other adults in your household relied on only a few kinds of low-cost food to feed the chi... = Often true

Or You or other adults in your household relied on only a few kinds of low-cost food to feed the chi... = Sometimes true

Or You or other adults in your household couldn't feed the child(ren) a balanced meal, because you c... = Often true

Or You or other adults in your household couldn't feed the child(ren) a balanced meal, because you c... = Sometimes true

Q19.8 The child(ren) were not eating enough because you and other adult members of the household just couldn't afford enough food. Was that often, sometimes or never true in the past 12 months?

- Often true (1)
- Sometimes true (2)
- Never true (3)
- Don't know (4)
- Prefer not to answer (5)

Display This Question:

If Which of the following statements best describes the food eaten in your household in the past 12... = Sometimes you and other household members did not have enough to eat

Or Which of the following statements best describes the food eaten in your household in the past 12... = Often you and other household members didn't have enough to eat

Or You and other household members worried that food would run out before you got money to buy more.... = Often true

Or You and other household members worried that food would run out before you got money to buy more.... = Sometimes true

Or The food that you and other household members bought just didn't last, and there wasn't any money... = Often true

Or The food that you and other household members bought just didn't last, and there wasn't any money... = Sometimes true

Or You and other household members couldn't afford to eat balanced meals. In the past 12 months was... = Often true

Or You and other household members couldn't afford to eat balanced meals. In the past 12 months was... = Sometimes true

Or You or other adults in your household relied on only a few kinds of low-cost food to feed the chi... = Often true

Or You or other adults in your household relied on only a few kinds of low-cost food to feed the chi... = Sometimes true

Or You or other adults in your household couldn't feed the child(ren) a balanced meal, because you c... = Often true

Or You or other adults in your household couldn't feed the child(ren) a balanced meal, because you c... = Sometimes true

Q19.9 In the past 12 months, since last April did you or other adults in your household ever cut the size of your meals or skip meals because there wasn't enough money for food?

- Yes (1)
- No (2)
- Don't know (3)
- Prefer not to answer (4)

Display This Question:

If In the past 12 months, since last April did you or other adults in your household ever cut the si... = Yes

Q19.10 How often did this happen?

- Almost every month (1)
- Some months but not every month (2)
- Only 1 or 2 months (3)
- Don't know (4)
- Prefer not to answer (5)

Display This Question:

If Which of the following statements best describes the food eaten in your household in the past 12... = Sometimes you and other household members did not have enough to eat

Or Which of the following statements best describes the food eaten in your household in the past 12... = Often you and other household members didn't have enough to eat

Or You and other household members worried that food would run out before you got money to buy more.... = Often true

Or You and other household members worried that food would run out before you got money to buy more.... = Sometimes true

Or The food that you and other household members bought just didn't last, and there wasn't any money... = Often true

Or The food that you and other household members bought just didn't last, and there wasn't any money... = Sometimes true

Or You and other household members couldn't afford to eat balanced meals. In the past 12 months was... = Often true

Or You and other household members couldn't afford to eat balanced meals. In the past 12 months was... = Sometimes true

Or You or other adults in your household relied on only a few kinds of low-cost food to feed the chi... = Often true

Or You or other adults in your household relied on only a few kinds of low-cost food to feed the chi... = Sometimes true

Or You or other adults in your household couldn't feed the child(ren) a balanced meal, because you c... = Often true

Or You or other adults in your household couldn't feed the child(ren) a balanced meal, because you c... = Sometimes true

Q19.11 In the past 12 months, did you (personally) ever eat less than you felt you should because there wasn't enough money to buy food?

- Yes (1)
- No (2)
- Don't know (3)
- Prefer not to answer (4)

Display This Question:

If Which of the following statements best describes the food eaten in your household in the past 12... = Sometimes you and other household members did not have enough to eat

Or Which of the following statements best describes the food eaten in your household in the past 12... = Often you and other household members didn't have enough to eat

Or You and other household members worried that food would run out before you got money to buy more.... = Often true

Or You and other household members worried that food would run out before you got money to buy more.... = Sometimes true

Or The food that you and other household members bought just didn't last, and there wasn't any money... = Often true

Or The food that you and other household members bought just didn't last, and there wasn't any money... = Sometimes true

Or You and other household members couldn't afford to eat balanced meals. In the past 12 months was... = Often true

Or You and other household members couldn't afford to eat balanced meals. In the past 12 months was... = Sometimes true

Or You or other adults in your household relied on only a few kinds of low-cost food to feed the chi... = Often true

Or You or other adults in your household relied on only a few kinds of low-cost food to feed the chi... = Sometimes true

Or You or other adults in your household couldn't feed the child(ren) a balanced meal, because you c... = Often true

Or You or other adults in your household couldn't feed the child(ren) a balanced meal, because you c... = Sometimes true

Q19.12 In the past 12 months, were you (personally) ever hungry but didn't eat because you couldn't afford enough food?

- Yes (1)
- No (2)
- Don't know (3)
- Prefer not to answer (4)

Display This Question:

If Which of the following statements best describes the food eaten in your household in the past 12... = Sometimes you and other household members did not have enough to eat

Or Which of the following statements best describes the food eaten in your household in the past 12... = Often you and other household members didn't have enough to eat

Or You and other household members worried that food would run out before you got money to buy more.... = Often true

Or You and other household members worried that food would run out before you got money to buy more.... = Sometimes true

Or The food that you and other household members bought just didn't last, and there wasn't any money... = Often true

Or The food that you and other household members bought just didn't last, and there wasn't any money... = Sometimes true

Or You and other household members couldn't afford to eat balanced meals. In the past 12 months was... = Often true

Or You and other household members couldn't afford to eat balanced meals. In the past 12 months was... = Sometimes true

Or You or other adults in your household relied on only a few kinds of low-cost food to feed the chi... = Often true

Or You or other adults in your household relied on only a few kinds of low-cost food to feed the chi... = Sometimes true

Or You or other adults in your household couldn't feed the child(ren) a balanced meal, because you c... = Often true

Or You or other adults in your household couldn't feed the child(ren) a balanced meal, because you c... = Sometimes true

Q19.13 In the past 12 months, did you (personally) lose weight because you didn't have enough money for food?

- Yes (1)
- No (2)
- Don't know (3)
- Prefer not to answer (4)

Display This Question:

If The child(ren) were not eating enough because you and other adult members of the household just c... = Often true

Or The child(ren) were not eating enough because you and other adult members of the household just c... = Sometimes true

Or In the past 12 months, since last April did you or other adults in your household ever cut the si... = Yes

Or In the past 12 months, did you (personally) ever eat less than you felt you should because there... = Yes

Or In the past 12 months, were you (personally) ever hungry but didn't eat because you couldn't affo... = Yes

Or In the past 12 months, did you (personally) lose weight because you didn't have enough money for... = Yes

Q19.14 In the past 12 months, did you or other adults in your household ever not eat for a whole day because there wasn't enough money for food?

- Yes (1)
- No (2)
- Don't know (3)
- Prefer not to answer (4)

Display This Question:

If In the past 12 months, did you or other adults in your household ever not eat for a whole day bec... = Yes

Q19.15 How often did this happen?

- Almost every month (1)
- Some months but not every month (2)
- Only 1 or 2 months (3)
- Don't know (4)
- Prefer not to answer (5)

Display This Question:

If Are there children under 18 in your household? = Yes

And The child(ren) were not eating enough because you and other adult members of the household just c... = Often true

Or The child(ren) were not eating enough because you and other adult members of the household just c... = Sometimes true

Q19.16 In the past 12 months, did you or other adults in your household ever cut the size of any of the children's meals because there wasn't enough money for food?

- Yes (1)
- No (2)
- Don't know (3)
- Prefer not to answer (4)

Display This Question:

If Are there children under 18 in your household? = Yes

And The child(ren) were not eating enough because you and other adult members of the household just c... = Often true

Or The child(ren) were not eating enough because you and other adult members of the household just c... = Sometimes true

Q19.17 In the past 12 months, did any of the children ever skip meals because there wasn't enough money for food?

- Yes (1)
- No (2)
- Don't know (3)
- Prefer not to answer (4)

Display This Question:

If In the past 12 months, did any of the children ever skip meals because there wasn't enough money... = Yes

Q19.18 How often did this happen?

- Almost every month (1)
- Some months but not every month (2)
- Only 1 or 2 months (3)
- Don't know (4)
- Prefer not answer (5)

Display This Question:

If Are there children under 18 in your household? = Yes

And The child(ren) were not eating enough because you and other adult members of the household just c... = Often true

Or The child(ren) were not eating enough because you and other adult members of the household just c... = Sometimes true

Q19.19 In the past 12 months, were any of the children ever hungry but you just couldn't afford more food?

- Yes (1)
- No (2)
- Don't know (3)
- Prefer not to answer (4)

Display This Question:

If Are there children under 18 in your household? = Yes

And The child(ren) were not eating enough because you and other adult members of the household just c... = Often true

Or The child(ren) were not eating enough because you and other adult members of the household just c... = Sometimes true

Q19.20 In the past 12 months, did any of the children ever not eat for a whole day because there wasn't enough money for food?

- Yes (1)
- No (2)
- Don't know (3)
- Prefer not to answer (4)

End of Block: CCHS Household Food Security Survey

Start of Block: Conclusion

Q20.1 Thank you so much for your participation in our study. Your contribution will help us to better understand how LGBTQ2SIA+ Torontonians are affected by the pandemic and could help us to develop recommendations for policies that support and benefit marginalized members of our community.


Thank you for your participation!

End of Block: Conclusion
